# The PrEPARE Pretoria Project: protocol for a cluster-randomized factorial-design trial to prevent HIV with PrEP among adolescent girls and young women in Tshwane, South Africa

**DOI:** 10.1186/s12889-020-09458-y

**Published:** 2020-09-15

**Authors:** Wendee M. Wechsberg, Felicia A. Browne, Jacqueline Ndirangu, Courtney Peasant Bonner, Alexandra M. Minnis, Laura Nyblade, Ilene S. Speizer, Brittni N. Howard, Bronwyn Myers, Khatija Ahmed

**Affiliations:** 1 Substance Use, Gender, and Applied Research Program, RTI International, 3040 E. Cornwallis Road, Research Triangle Park, NC 27709-2194 USA; 2 Maternal and Child Health, University of North Carolina Gillings School of Global Public Health, 135 Dauer Drive, Chapel Hill, NC 27599 USA; 3 Department of Psychology, North Carolina State University, 640 Poe Hall, Campus Box 7650, Raleigh, NC 27695 USA; 4 Psychiatry and Behavioral Sciences, Duke University School of Medicine, 40 Duke Medicine Circle, Durham, NC 27710 USA; 5 Health Behavior, University of North Carolina Gillings School of Global Public Health, 135 Dauer Drive, Chapel Hill, NC 27599 USA; 6 Center for Global Health, RTI International, 2150 Shattuck Avenue, Suite 800, Berkeley, CA 94704 USA; 7 Epidemiology Division, Berkeley School of Public Health, University of California, 2121 Berkeley Way, Room 5302, Berkeley, CA 94720 USA; 8 International Global Health Division, International Development Group, RTI International, 701 13th Street NW #750, Washington, DC 20005 USA; 9 Alcohol and Drug Abuse Research Unit, Medical Research Council Francie van Zijl Drive, Parow Valley, Cape Town, South Africa; 10 Setshaba Research Centre, 2088 Block H, Soshanguve, 0152 South Africa

**Keywords:** HIV, Sexual and reproductive health (SRH), Stigma, Health Policy Project Stigma and Discrimination-reduction training (HPP S&D), Clinic staff, Young Women’s Health CoOp (YWHC), Alcohol and drug use, Gender-based violence, Adaptation

## Abstract

**Background:**

Despite increased prevention efforts, HIV remains the leading cause of death among adolescent girls and young women in South Africa. Although research indicates important determinants of HIV acquisition at the individual and interpersonal levels, structural-level stigma and discrimination continue to be critical barriers to reaching and retaining this key population for HIV prevention and sexual and reproductive health services. Innovative and multilevel interventions are needed that can address the intersectional structural and gender issues that young women face, including stigma, alcohol and drug use, gender-based violence, and other risk factors when seeking health services. Oral pre-exposure prophylaxis (PrEP) taken daily has been found to be an effective biomedical HIV prevention tool. Testing a comprehensive gender-focused biobehavioral HIV prevention intervention that is inclusive of social ecological determinants, such as stigma and discrimination reduction in clinics, is critical for reducing HIV among adolescent girls and young women.

**Methods:**

This project involves both a Community Collaborative Board and a Youth Advisory Board in helping to adapt the Young Women’s Health CoOp intervention and the Health Policy Project (HPP) Stigma and Discrimination (S&D) reduction training curriculum to the setting and population. This study uses a two-by-two factorial design with stratified randomization of 12 clinics, each with distinct catchment areas. The Young Women’s Health CoOp addresses substance use, sexual risk, violence prevention and sexual negotiation, condom demonstration, and problem solving with the following additions: knowledge of PrEP, the importance of PrEP adherence, and sexual and reproductive health. Adolescent girls and young women will be assessed with behavioral and biological measures at baseline, 3-, 6- and 9-month follow-up. The S&D reduction training is provided for all staff in the clinics randomized to this condition. Clinic staff will be surveyed at baseline, 4- and 8-month follow-up. We will recruit 900 AGYW from communities in the 12 clinic catchment areas.

**Discussion:**

The study findings, if efficacious across the outcomes, will be incorporated into the gender-focused HIV prevention intervention toolkit and disseminated to inform multilevel prevention approaches.

**Trial registration:**

ClinicalTrials.gov. Identifier: NCT04048551 (Recruiting). Registered: August 7, 2019 (Retrospectively registered).

## Background

Despite important progress globally to reduce HIV incidence, adolescent girls and young women (AGYW) continue to be disproportionately at risk of HIV acquisition [1]. In South Africa, new HIV infections are concentrated among AGYW between 15 to 24—this group accounts for over 25% of new in-country infections, three times the rate of young men in this age group [2]. Additionally, estimates suggest that in some areas of South Africa, AGYW have an HIV prevalence of 4%, which increases to 24% for young women aged 20 to 24 [2]. This highlights the high incidence of HIV in late adolescence and early adulthood and the critical need for prevention in this age group.

Research indicates important determinants of HIV acquisition at the individual and interpersonal levels. However, structural-level stigma and discrimination (S&D) continues to be a critical barrier to reaching and retaining this key population for HIV prevention and sexual and reproductive health (SRH) services [3–6]. AGYW who engage in high-risk sex are aware of their risk and desire healthcare services, but service access is often impeded by poor treatment by clinic staff [6, 7]. In formative focus group discussions conducted in Tshwane for this study, AGYW expressed concerns about treatment by nurses when seeking SRH services and about confidentiality in clinics [6, 7]. S&D also prevents engagement and retention in HIV prevention services [8–12]. Anticipated and experienced HIV stigma extends to biomedical HIV prevention strategies, such as pre-exposure prophylaxis (PrEP), and is also a barrier to HIV prevention efforts [13–16]. Providing PrEP in clinics may not be effective if vulnerable AGYW are disinclined to visit clinics because of fear of S&D or patronizing treatment by clinic staff based on assumptions about their age and sexual activity. Consequently, S&D training for clinic staff is essential to reduce barriers for AGYW to access services.

Intersectional stigma toward AGYW is the convergence of multiple stigmatized identities, such as age, health, gender, behavior, and socioeconomic status [17]. Because individuals’ characteristics/identities are not in isolation, stigma needs to be addressed through multiple-level combination interventions [18]. Further, intersectional issues—including condomless sex and the lack of personal power or skills to negotiate safer sex, the need to conduct transactional sex, cross-generational sex, gender-based violence (GBV), substance use, and other risky behaviors—affect the likelihood that AGYW may acquire HIV during emerging adulthood [2, 19].

Globally, momentum is increasing to avert HIV infection among AGYW via the use of comprehensive strategies in the care continuum. The South African government acknowledges this need, as demonstrated by its involvement in the Determined, Resilient, Empowered, AIDS-free, Mentored and Safe (DREAMS) program, which aims to prevent HIV among vulnerable AGYW by empowering them through social asset building and by providing post-violence care, contraception through SRH services, and HIV prevention such as condoms and biomedical prevention such as PrEP [20].

### Oral PrEP: a female-controlled HIV prevention tool

Oral PrEP, a combination of the antiretroviral (ARV) medications emtricitabine (FTC) and tenofovir disoproxil fumarate (TDF) taken daily, prevents HIV acquisition among individuals who are HIV-negative. It has been found to be effective among women in Africa [21, 22]. It was approved for use by the U.S. Food and Drug Administration in 2012 [23] and the South African Government in 2015 [24].

Because of an inability to negotiate condom use or mutual monogamy [25–27], many AGYW are unable to protect themselves from HIV, even when they are particularly vulnerable to HIV transmission [2]. These AGYW may benefit from PrEP because it does not require partner involvement. Recent open-label demonstration projects that offered oral PrEP to young women [28], including some in South Africa, have demonstrated that PrEP is feasible among this age group [29]. However, placebo-controlled efficacy trials, such as the FemPrEP and VOICE trials found low PrEP adherence rates [30–32]. Although oral PrEP offers a promising HIV prevention strategy for AGYW, its effectiveness depends on high adherence [33]. To support PrEP uptake and adherence, an integrated HIV prevention program is needed that addresses multiple barriers and risk behaviors, with a focus on the individual, interpersonal, and structural levels, that may impede PrEP readiness, uptake, adherence, and engagement in safer sexual behaviors among AGYW who are HIV-negative [34].

### The adapted Young Women’s Health CoOp

The Women’s Health CoOp (WHC), the original intervention, is an evidence-based woman-focused HIV prevention intervention grounded in empowerment and feminist theory to increase individual knowledge and skills to reduce substance use and increase personal power around HIV risk behaviors [35]. The WHC has been shown to be efficacious in several National Institutes of Health-funded studies in South Africa [36–39].

The Young Women’s Health CoOp (YWHC) was originally adapted from a U.S. adolescent project and subsequently fielded in South Africa for a younger generation of women at risk for HIV [4, 19, 40]. The YWHC included the core elements of the most recent WHC addressing substance use, sexual risk, violence prevention and sexual negotiation, condom demonstration, and problem solving, including role-play practice. The next generation of the YWHC, which was adapted for the current study entitled, The *PRevention, Empowering, and PRotEcting* (PrEPARE) Project includes knowledge of PrEP, such as the importance of adherence and in-depth information on contraception and other SRH options. The added elements of PrEP and SRH material, along with assistance with accessing clinics for PrEP initiation and adherence and contraception, ensures that AGYW receive a more comprehensive HIV prevention program and SRH services.

Although the YWHC addresses the individual and interpersonal needs of AGYW who engage in risk behaviors, such as alcohol and drug use and condomless sex, it did not address the structural barriers, such as stigma and discrimination, that many AGYW face when accessing clinics for services. Consequently, the structural aim of this study addresses S&D-reduction training in clinics.

### Addressing structural stigma and discrimination in clinics

The Health Policy Project (HPP) Health Facility HIV-Stigma and Discrimination reduction training curriculum addresses barriers that arise from S&D. The HPP curriculum is based on a decade of implementation experience in Africa, South and South-East Asia, and the Caribbean [41]. It has been further adapted and implemented in a range of settings, most recently in Ghana [42], Tanzania [43, 44] and other regions of the world [45, 46]. The curriculum provides participatory training modules that address three key actionable drivers of HIV stigma: lack of understanding of S&D; fear of workplace HIV transmission; and clinic staff attitudes. For this study, the HPP curriculum was modified to address the lives and experiences of AGYW seeking SRH and PrEP services.

To decrease structural S&D in clinics and increase AGYW’s agency to protect themselves, a multilevel strategy is needed. The present study seeks to determine whether implementing a multilevel, woman-focused intervention for PrEP readiness, uptake, and adherence among vulnerable AGYW in Tshwane, South Africa, that includes S&D reduction assessment and training in clinics, is a viable complement to South Africa’s National Strategic plan for HIV, which includes a focus on AGYW [47]. It also seeks to address barriers to accessing SRH services among this vulnerable population by reducing health facility-wide S&D.

## Methods/design

### Aim and objectives

This project comprises three specific aims. Aim 1, which was completed during the study’s formative phase, involved identifying and randomizing eligible clusters (study clinics) and engaging community stakeholders through a Community Collaborative Board (CCB) and Youth Advisory Board (YAB) to assist in the adaptation of the YWHC and S&D reduction training program to the South African geographic and cultural context. This study protocol is based on Aims 2 and 3, the experimental phase of the study. Aim 2 is to evaluate the impact of the HPP S&D reduction training among clinic staff on the use of HIV and SRH services by AGYW, including PrEP, and staff attitudes and behaviors toward AGYW at 4- and 8-month follow-up. Aim 3 is to test the efficacy of a multilevel HIV prevention strategy that addresses structural (S&D reduction), interpersonal (peer social support), and individual (personal agency, substance use, and GBV) factors on PrEP readiness, uptake, and adherence; HIV status; and other risk-taking behaviors at 3-, 6-, and 9-month follow-up among vulnerable AGYW. We plan to recruit 900 AGYW across 12 communities in clinic catchment areas for the experimental phase of study.

### Setting

We conducted feasibility assessments of potential study sites in provincial and city clinics in Tshwane, South Africa; 15 clinics were selected as the final study clinics. Each assessment included engaging the clinic manager and requesting permission to conduct the assessment. The selected clinics had (1) community ward-based outreach teams (WBOTs), (2) comprehensive SRH services, and (3) designated youth-friendly nurses and/or services. Other criteria included (a) the jurisdiction of the clinic—city or provincial, as this determines the allocation of resources and infrastructure, (b) reported average number of AGYW visiting the clinic per month, and (c) clinic location (peri-urban or semi-rural community) within the Tshwane district. As a result, the final sample consisted of 12 study clinics, 8 provincial clinics and 4 city clinics, with 3 additional clinics as backups if one or more of the selected clinics turns out not to be viable.

### Study design

**Fig. 1:**
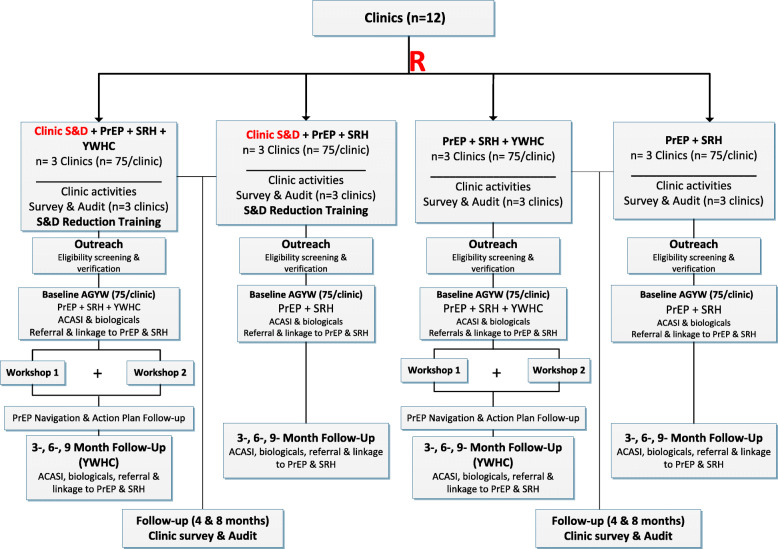
Study Design

This cluster randomized trial utilizes a 2 × 2 factorial design [48] to assess the impact of S&D reduction training and the YWHC intervention, resulting in four study conditions: (1) Clinic S&D reduction training + PrEP/SRH + YWHC, (2) Clinic S&D reduction training + PrEP/SRH only, (3) No Clinic S&D reduction training +PrEP/SRH + YWHC, (4) No Clinic S&D reduction training + PrEP/SRH only (Fig. 1).

### Randomization

Stratified randomization was conducted via SAS Software after clinic selection was finalized. The sample of 12 clinics was first grouped into three strata (peri-urban/city, peri-urban/provincial, and semi-rural/provincial) based on geographic location type (peri-urban vs. semi-rural) and clinic administration type/jurisdiction (city vs. provincial), with four clinics in each stratum. Within each stratum, clinics were then randomly assigned to study conditions based on a 1:1:1:1 allocation. Because there may be important differences pertinent to the outcomes that cannot be made homogenous, such as geographical region, stratified randomization was conducted to achieve even distribution of these factors across study arms. This reduces variability in the estimation of the intervention effect, with resulting increases in power/precision of estimates [49].

The six clinics randomized to receive the S&D reduction training will participate in workshops aimed to reduce clinic staff’s S&D attitudes and behaviors toward AGYW. For the 6 clinics randomized to the YWHC, AGYW participants recruited from communities in clinic catchment areas will be asked to participate in two workshops.

### Study procedures

#### Data collection: clinic level

At baseline, all clinics (those receiving training and those receiving no training) complete a clinician (e.g., physicians, nurses) or clinic support staff (e.g., receptionists, clerks) survey assessing (after providing informed consent) SRH knowledge and service provision, attitudes toward PrEP and AGYW seeking PrEP, and observations of stigmatizing and discriminatory behavior in their clinic toward AGYW seeking services for SRH, HIV, and PrEP (see Clinician Questionnaire & Support Staff Questionnaire in Supplementary Files 1 & 2). This survey is re-administered at 4- and 8-months post baseline. Given the staff turnover and availability and to ensure to anonymity, staff surveys are not linked across time points; consequently, changes at the clinic level are assessed. Clinic support staff surveys have been translated into a local language, Setswana, for easier comprehension. A clinical audit is also being conducted at baseline and 4-month and 8-month follow-up by research study staff to collect information on service utilization, including the number of AGYW receiving health services (including HIV, birth control, and antenatal care) from clinical records; PrEP prescriptions from pharmacy and study nurse records; availability of birth control methods; presence of clinic guidelines and standards of practice for serving sexually active AGYW; working hours that are convenient for AGYW, as well as adequate staff to provide youth friendly services. This audit is conducted across all study clinics.

#### Stigma and discrimination reduction training

After baseline data collection, marketing and planning, including the introduction of the training, are proposed for clinics randomized to receive the S&D reduction training so that time is allocated and protected for staff to attend the workshops. Although all clinic staff members are eligible and encouraged to attend, this study relies on clinic managers to approve who, among those interested, can take part in the assessments and training, as some clinic operations need to be maintained during this time. The HPP S&D reduction curriculum encourages all staff at all levels to be involved in the training workshops [41] because they are likely to interact with AGYW. For example, in South Africa, support staff such as receptionists and clerks often interact with AGYW because they serve as gatekeepers to clinicians.

The S&D reduction training incorporates important baseline findings for relevance and ownership through participatory activities addressing key drivers of clinic stigma toward AGYW seeking PrEP and birth control. This includes building awareness of AGYW stigma—its causes, forms and manifestations—reflecting on one’s own stigmatizing experiences and attitudes towards AGYW and developing a plan/pledge with actionable steps to reduce S&D in the clinic. The HPP curriculum [41, 42] has been adapted with respect to time, target audience, and type of stigma relevant to this study. Modifications include examples of AGYW seeking services in pictorial displays and other pictures to elicit conversation in the workshops to address various types of bias (see Table 1 for an overview of the S&D reduction training).

**Table 1:** Overview of the S&D Reduction Training

**Workshop 1**	
1	Opening activities (warmup games, songs, energizers)
2	Naming stigma through pictures
3	Our experience as the stigmatizer and the stigmatized
4	Confidentiality and stigma
**Workshop 2**	
1	Breaking the sex ice
2	Fears about prescribing pre-exposure prophylaxis (PrEP) to adolescent girls and young women
**Workshop 3**	
1	Panel discussion with adolescent girls and young women
2	Challenge the stigma—and be the change!
3	The blame game: Things people say
4	Stigma-free services for adolescents and young people
5	Writing a code of practice and action plan

#### Implementation of stigma and discrimination training

The S&D reduction training workshops are conducted by clinic staff who have been selected and trained by experienced S&D reduction trainers to facilitate these workshops. Preferably, the appointed facilitators are clinic staff who work with youth, such as youth-friendly nurses, and are interested in implementing the S&D reduction training workshops. They are supported by YAB members and research study staff and observed by the S&D reduction master trainers. The clinic S&D reduction training has 6 modules, with added clinic wide booster training workshops to account for clinic staff turnover.

### Data collection: adolescent girls and young women level

#### Recruitment

AGYW are recruited in communities that are in the study clinic’s catchment area. Recruitment is conducted through research study staff, who may also be assisted by trained WBOTs associated with the study clinics. However, screening is only conducted and performed by research study staff. Recruitment is conducted via street outreach at identified areas where AGYW are known to frequent. Street outreach is an established recruitment method that has been used successfully in previous research studies [50–53], including Tshwane WHC studies to reach and recruit a high number of women most at risk of HIV. Study staff visit these hotspots regularly to establish a known presence in the community and gain rapport with community members. Recruitment also is conducted through referrals from the clinic or other staff who conduct HIV and/or pregnancy testing in the community, such as HIV and AIDS, STI, and TB (HAST) counselors.

Participant eligibility criteria include the following: (1) identify as female; (2) HIV negative status; (3) between ages 16 and 24 years; (4) have had condomless sex in the past 3 months with a male partner; (5) not currently pregnant and do not want to get pregnant within the next year; (6) interested in taking a daily pill to prevent HIV (PrEP); (7) not having previously participated in the formative phase of the study; (8) not previously or not currently participating in any other PrEP-related project or research study; (9) not previously or not currently participating in any other HIV study in Tshwane; (10) not on multidrug-resistant tuberculosis (MDR-TB) treatment; (11) lives in one of the target communities; (12) intends to stay in the Tshwane district for the next 12 months; (13) agrees to provide contact information; (14) willing to undergo rapid HIV testing; and (15) willing to undergo pregnancy testing.

Because of the eligibility requirement of an HIV-negative and nonpregnant status, interested potential participants must first consent to rapid testing for HIV and pregnancy to confirm eligibility. Additionally, co-enrollment in another HIV study is also checked and any individual who is currently enrolled is excluded.

#### Screening

Verbal consent is obtained to screen the potential participant and determine eligibility. Initial eligibility screening of potential participants is performed individually and in private by research study staff. After the initial screening, the next step is to confirm that the participant is HIV-negative, not pregnant, and not enrolled in another HIV study; this is done at the study clinic. Potential participants provide informed consent before conducting these tests. All participants receive a rapid HIV test and a pregnancy test. The only exception is if they have tested negative for HIV the same day and they have an acceptable proof of their HIV testing result, such as a clinic card. If a potential participant cannot be seen for the baseline appointment on the same day as initial screening, they are not tested for HIV and pregnancy until the day of their scheduled baseline appointment to minimize repeat testing. They are rescreened using the initial eligibility screening tool to ensure they meet study eligibility criteria on the day of enrollment.

#### Consent or assent

The intake process takes place within the compound of the study clinic, typically in an outdoor tent because of the limited physical space in clinics. Written informed consent or assent is obtained from eligible participants before study enrollment. For potential participants who are younger than 18, consent is required from their mother or a trusted adult woman at least 25 years old who may serve in loco parentis (“in place of a parent”). Parental waiver has been requested to protect a participant’s confidentiality if they are uncomfortable having their mother consent for them. This approach has been used successfully in our previous South African studies with adolescents [19, 51]. In loco parentis enables the participant to select a female adult (either identified by the participant themselves or by the study staff) to provide consent on her behalf.

Once the adult woman who will provide consent is identified, study staff schedule an appointment for the potential participant and the adult to sign the mother/in loco parentis consent. The potential participant is screened separately again and assented separately from the adult woman to prevent coercion, maintain confidentiality, and provide the opportunity for the participant to decline. The adult woman is also required to sign a confidentiality agreement form as part of the consent process so they understand that their experience at the project site is confidential and that they must maintain confidentiality regarding the person for whom they consented.

#### Intake assessment

After consenting/assenting, participants also sign a release of medical records permitting the release of information on their SRH, PrEP screening and monitoring results, and other referrals and care pertinent to the study.

Participants take a breathalyzer test to detect recent alcohol use (conducted earlier in the appointment because of the limited detection window for alcohol breath scans) and provide urine for drug screening, locator information is collected, and a photograph of the participant is taken to identify the participant for subsequent appointments. This photo is returned or shredded at the final appointment. The participant then completes a baseline questionnaire on a computer tablet via audio computer-assisted self-interview (ACASI) in either English or Setswana (see AGYW Health CoOp Questionnaire in Supplementary File 3). Trained field staff are available to assist the participant at any time and provide referrals based on prompts related to self-reported violence, symptoms of psychological distress, or suicidal ideation experienced in the past 3 months that are triggered as the participant completes the questionnaire. The questionnaire is derived from components of the youth-specific modification of the Revised Risk Behavior Assessment (RRBA) [54], which has been adapted and modified for many studies in South Africa. The modified RRBA for this study contains sections on PrEP knowledge, contraception and other SRH services access and utilization, sexual communication, STI symptoms, alcohol and other drug use, relationship equity and sexual control, economic dependence, personal agency, psychological distress, victimization, and peers and social support. After completing the questionnaire, urine drug screening results are provided to assess the recent use of amphetamine, methamphetamine, benzodiazepine, cocaine, marijuana, opioids, and MDMA. These screening tests are repeated at 3-, 6-, and 9-month follow-up assessments.

After the intake appointment, participants interested in initiating PrEP are referred to the youth-friendly nurse in their respective study clinic or the study’s roaming nurse to screen for PrEP eligibility using the National Department of Health (NDoH) guidelines. Baseline screening for PrEP involves reporting of no MDR-TB (part of the study eligibility criteria), HIV and pregnancy testing (conducted prior to the intake process to confirm HIV-negative and not pregnant), estimation of creatinine clearance, and hepatitis B virus (HBV) screening. Contraindications for PrEP use includes poor renal function (estimated creatinine clearance < 60 mL/min) and absence of the hepatitis B surface antigen and antibody. Presence of HBV is not a contraindication for PrEP; however, liver function monitoring is advised if PrEP is initiated. Participants not eligible for PrEP are linked to the public health clinic to receive the standard of care for each respective medical condition. Individuals who are not eligible for PrEP can still remain enrolled in the study.

Participants may decide to initiate PrEP at any time during the study. However, those who do not initiate PrEP during their baseline appointment have to repeat all PrEP eligibility screening. Participants who decide to initiate PrEP are counselled on the importance of adherence and using other protective strategies for up to 20 days of daily dosing before protective levels in vaginal tissue are achieved. Participants are also given guidance on proper management of mild side effects that may occur after initiation and are advised to continue to use condoms and birth control to protect themselves against other STIs and unplanned pregnancy, as PrEP does not provide this type of protection.

Clinic visits to test for HIV and pregnancy status are scheduled 1 month after initiation of PrEP and every 3 months thereafter. At 6 months post initiation of PrEP, screening of creatinine levels is conducted to estimate creatine clearance. If a participant tests positive for HIV at any point in the follow-up appointments, PrEP is stopped for that participant and an active referral is provided for test and treat according the South African HIV guidelines. Nonclinical behavioral study data collection follow-up visits continue to occur at 3, 6, and 9 months post enrollment regardless of whether or when PrEP is initiated or when a participant’s PrEP clinic visits take place.

#### Sexual and reproductive health services

All participants who complete their baseline appointment are actively referred to the designated youth-friendly nurse or youth-friendly service at their respective study clinic for SRH services; specifically, birth control services. Follow-up on these linkages is conducted at the 3-month follow-up appointment for participants in the control groups and during monthly check-ins for participants in the YWHC groups.

#### YWHC workshops

The YWHC workshops comprise a 2-session, 4-module program designed to build on the nexus of substance use, HIV, and GBV by increasing knowledge, skills, and agency to reduce GBV and substance use, and ways to decrease sexual risk and HIV incidence. Previous research in South Africa has shown positive outcomes with the WHC, including that women in the WHC were more likely to use condoms with their boyfriends during their last episode of sexual intercourse, more likely to use female condoms with their boyfriends in the past month, more likely to negotiate condom use, and less likely to report daily substance use at follow-up than women who were not in the WHC [38, 39, 52]. The workshops also include voices and quotes from the formative focus group discussion participants (see Table 2 for an overview of the workshops). Typically, workshops are conducted approximately 7 to 10 days apart, but can be completed on the same day and in either order because of limited availability of the participants; for example, some participants are full-time students. Both workshops end with participants developing an individualized and personalized risk-reduction action plan with assistance from staff. Also, any relevant referrals are offered. Participants also receive toiletry kits and pill boxes with a colorful bag to discreetly store their PrEP. Once both workshops are completed, participants complete a Satisfaction Form to provide feedback and suggestions regarding the intervention. Staff conduct further follow-up with participants individually to check in on their personal action plans at least monthly via mobile phone or in person.

**Table 2:** Overview of the YWHC Workshops

**Workshop 1**	
**Topics**	
• Becoming an adult woman and influences	
• Sex, sexual expectations, and risk	
• Our reproductive bodies (female and male anatomy)	
• STIs and HIV	
• Ways to reduce risk	
• Male and female condoms	
• Ways of communicating, negotiating, and problem-solving	
• PrEP	
• Birth control	
• Action plan (complete Workshop 1 goals and steps in workshop booklet)	
**Workshop 2**	
**Topics**	
• Inequality and gender power	
• Becoming strong women and concern for how boyfriends treat you	
• Abuse and violence	
• Safety tips for going out	
• Alcohol and drug use	
• Parenting	
• The importance of education and goals	
• Social support, especially taking PrEP	
• Action plan (complete Workshop 2 goals and steps in workshop booklet)	

#### PrEP navigation after PrEP initiation

Although the South African National Strategic Plan for HIV, TB and STIs (2017–2022) [47] outlines the availability of PrEP for all AGYW who are HIV-negative and at risk of HIV, PrEP roll-out in public health clinics has yet to occur. Consequently, a majority of public health clinic staff have not been trained on PrEP prescribing and dispensing protocols. Prior to the national roll-out, the PrEPARE Project partnered with the NDoH to train youth-friendly nurses and pharmacists charged with supporting and implementing PrEP delivery from all study clinics on standardized PrEP protocols. A roaming nurse has been hired to assist in the management and support of dispensing PrEP where there is a shortage of trained clinic staff.

PrEP navigation, which occurs through phone calls and text messaging, provides participants in the YWHC intervention arms who are on PrEP with much-needed support, specifically in the first 1 to 2 weeks of PrEP initiation where many participants may experience minor side effects that often lead to PrEP discontinuation. Study staff work with participants to develop feasible plans that can support the daily use of PrEP, such as the pill boxes they receive, adherence strategies, social support, and addressing other concerns about PrEP. Participants also are reminded about their upcoming refill and check-up appointments. This navigation occurs throughout the study duration as participants are due for their refills.

#### Follow-up assessments

Participants in both study arms return for their 3-, 6- and 9-month behavioral and biological follow-up assessments. Research staff track participants using locator information provided during the intake appointment to help retain participants. The follow-up visit includes reconsenting; updating locator information; a follow-up questionnaire via ACASI; and biological testing for HIV, pregnancy, alcohol use (breathalyzer), and other recent drug use. Dried blood spots (DBS) also are collected at these appointments from participants who report to be on PrEP. The PrEP medication used for this study is a combination of FTC 200 mg/TDF 300 mg tablets. The DBS are used to assess the presence of tenofovir diphosphate (TFV-DP), a measure of cumulative and recent adherence, using a previously validated methodology [55].

#### Data management and quality assurance

To protect confidentiality, the study assigns each participant a unique alphanumeric study participant identification number (PID). This PID is the only link between the behavioral and biological data and the identifying information collected for locating participants for their follow-up interviews. Locator forms, consent/assent agreements, and any data that can be linked through the PID are stored separately from other data in double-locked file cabinets in locked rooms at the study’s project site, with restricted access.

Data collection for this study is conducted by highly trained staff from the community who develop a rapport with the study participants to engender trust and elicit the most accurate data possible. All staff sign a confidentiality agreement and are trained on the study’s Quality Assurance Protocol and Quality Management Plan (QMP). Study data are encrypted before they are transmitted daily from the field site to secure servers in the United States. The US-based data manager reviews additional automated quality control checks that the software generates each day. If any critical inconsistencies are noted, the data manager contacts the project director and the field supervisor to resolve these inconsistencies. The Principal Investigator, other members of the research team, and the field staff receive daily field activity reports.

#### Data and safety monitoring plan and data and safety monitoring board

Procedures have been put in place to address adverse events (AEs) or serious adverse events (SAEs), such as improper disclosure of information or mental or emotional discomfort. As specified in our Data and Safety Monitoring Plan (DSMP), SAEs are reported to the Principal Investigator and the South African Co-Investigator/Medical Director within 24 h of an occurrence and to the Data and Safety Monitoring Board (DSMB), the funding agency, and the Institutional Review Board within 72 h, with appropriate action taken immediately. The study does not interfere with any activities or reports that are part of the public health clinics’ standard operating procedures that do not directly affect the study participants adversely. The DSMP ensures that procedures have been set in place to safeguard the security, validity, and integrity of study data, and that study staff are trained on the policies and procedures for data management according to the Quality Assurance Protocol and QMP. The DSMP also outlines the data analysis plan including preliminary analyses of data for quality assurance and to track the progress of the study.

The study established a DSMB comprising three members: a psychologist, an infectious disease clinician, and a bioethicist. The DSMB will meet every 6 months during the trial to review study progress and ensure adherence to the DSMP. This board is independent from the researchers and the study sponsoring institution. Board members discussed whether stopping rules were necessary for this study; they determined that stopping rules were not necessary.

#### Outcomes

The clinic-level primary outcomes for this study include clinic staff attitudes and environment, observed discrimination, and stigmatizing avoidance behaviors toward AGYW, as measured by staff surveys and the clinic audits. The AGYW-level primary outcomes include the level of PrEP readiness, uptake, and adherence (as measured by self-report and DBS), and SRH uptake. The AGYW-level secondary outcomes include frequency of substance use, as measured by the RRBA and biological drug screening and breathalyzer tests to assess recent alcohol use; GBV, assessed through self-reported experiences of emotional, physical and sexual abuse; sexual risk as measured by condomless sex, impaired sex, other sex partners; self-reported frequency of experienced stigma.

#### Sample size and power

For the AGYW analyses, the total sample size and number of clusters (clinic catchment areas) were selected to ensure sufficient power to detect meaningful differences in our primary outcomes, while also balancing considerations tied to reducing possible contamination (exposure to the intervention in control clusters) and implementation feasibility with a set number of clinics in the study area. Sample size estimates for tests of two proportions in a cluster-randomized design [56] were conducted for a 2-sided test with significance level of 0.05, power of .80, and intra-cluster correlation of 0.01 (based on our team’s past research in Cape Town communities), assuming 10% attrition over the 9-month follow-up period [52, 57]. This was not adjusted for multiple comparisons. Based on these parameters and calculations, the study sample includes 12 clusters (defined as clinics and their catchment areas), with a total sample size of 900 AGYW enrolled, 75 per cluster (clinic catchment area). This sample was chosen to ensure that meaningful differences in the primary outcomes of PrEP readiness, uptake, and adherence between groups would be detectable. We are powered to detect a difference between 9 to 13% in primary outcomes. Power analyses were first conducted in PASS software [58] and were refined in Stata [59] (see PrEPARE Additional Power Calculation Information in Supplementary File 4).

#### Analysis

Analysis of primary outcome measures will determine whether the provision of S&D reduction training for clinic staff and the YWHC intervention for AGYW will increase PrEP readiness, PrEP uptake, and PrEP adherence, and SRH uptake.

#### Clinic level

AGYW service utilization will be assessed and analyzed through clinic level data at each of the 12 study clinics during an 8-month period pre- and post-training (e.g., HIV testing; number of PrEP prescriptions). Baseline and follow-up clinic staff surveys will be analyzed to examine changes in attitudes and stigma at the clinic level. Initial analyses will be descriptive and evaluate group differences comparing intervention and control clinics using unpaired t-tests with unequal variance or nonparametric tests, as appropriate. Using multilevel modeling approach, we will investigate and account for clustering at the clinic level. We will use difference-in-differences (DD) methods as done in another South African study to examine utilization of services by AGYW and staff attitudes and behaviors in the 8 months between intervention and control clinics [60]. The DD estimator reflects the average change in clinics that received S&D reduction training, after the average change in utilization in control clinics is subtracted, assuming parallel trends in clinics over time had the S&D reduction training not been implemented. DD estimates will be calculated for each outcome separately; models will include covariates for time (pre- vs. post-intervention), treatment (training vs. no training) and an interaction between the two. The clinics identified are similar in size, services, and populations served. Any clinic features for which we cannot achieve balance through the design will be included as covariates in the multivariable DD analyses. A stigma score will constitute one primary outcome for these analyses. To examine short-term changes between pre-intervention and 4-month follow-up, DD methods can be used to compare the average change in stigma scores in clinics that received S&D reduction training, removing any changes observed in control clinics. We will use generalized estimating equations (GEE) [61] to examine provider-level stigma scores measured at 4- and 8-month follow-up comparing trained and untrained clinics, accounting for clustering at the clinic-level.

#### AGYW level

The testing of Aim 3 will be done by intention to treat analysis, examining the effects of intervention groups at both levels (clinic and individual) on PrEP readiness, uptake and adherence. PrEP uptake will be defined as any use of PrEP. We will consider both short-term uptake within the first 3 months after study enrollment and uptake at any point during the 9-month follow-up period. Initially, we will calculate the proportion of AGYW with any use of PrEP, overall, and within each study arm, calculating 95% confidence intervals using the binomial distribution. Given the need to consider the influence of the cluster randomization by clinic, we will use generalized estimating equations (GEE) [61] models with a logit link and an exchangeable correlation structure to estimate the effects of randomization groups on both short-term (3-month follow-up) and long-term follow-up period (9-month follow-up). Models will include both intervention groups (i.e., S&D reduction training and YWHC) as covariates. Effects of clustering by randomization clinic may be examined by adjusting the variance by the inflation factor [1 + (m-1)r], where *m* is the average clinic size and *r* is the interclass correlation estimate. If the Hausman assumption of correlation between the random and fixed effects is violated, then we may include fixed effects representing cluster identification.

Finally, we will include additional baseline covariates in the models, including sociodemographic factors and behavioral risks, should initial descriptive analyses suggest differences in the distribution of these factors across study arms. In subsequent exploratory analyses, we will examine the potential mediating and moderating roles of key behavioral, social, and structural factors hypothesized to influence PrEP uptake (e.g., social support; economic dependence), as outlined in Outcomes. To assess adherence in the past month, we will measure drug-level concentration, TFV-DF assessed through DBS samples, at 3, 6 and 9 months. The threshold for adherence will be established based on recommendations from ongoing studies and research designed to inform thresholds in women. Secondary adherence measures will include self-reported use, which will be examined as the number of days in the past 30 days when PrEP was taken. Using clinical/pharmacy data on PrEP dispensation and self-report of months in which PrEP was used, we will examine adherence (the number of months of PrEP use) and discontinuation. Initially, we will examine differences in the proportions of AGYW who are adherent at months 3, 6, and 9 in each study arm, calculating confidence intervals using a binomial distribution. Our analytic approaches will be similar to those described for uptake. We will use generalized linear mixed models to allow for multiple variance components, including clustering of clinics and repeated measures of adherence within individuals over time. We will use logit, Poisson, and linear regression approaches, as appropriate.

Analyses of secondary outcomes will use similar analytic approaches and examine hypotheses tied to intervention effects on behavioral risk endpoints (e.g., substance use) and on AGYW’s assessment of stigma (e.g., participants attending the S&D reduction trained clinics will report lower levels of stigma from clinic staff than participants who attend the non-trained clinics). Other model specifications, such as zero-inflated Poisson or negative binomial will be considered for count outcomes (e.g., the number of condomless sex acts) that have excess zero values or overdispersion.

From the AGYW outcomes, it is hypothesized that AGYW enrolled from clinics who received clinic-level S&D reduction training will have higher PrEP and SRH utilization than AGYW enrolled from clinics that did not receive training. AGYW enrolled from clinics that are randomized to receive the YWHC intervention will have higher PrEP readiness, uptake and adherence than those enrolled from clinics that are randomized to PrEP and SRH provision only. AGYW enrolled from communities in clinic catchment areas that are randomized to receive the YWHC will report less HIV-related risk at follow-up (e.g., GBV, substance use, condomless sex) than those enrolled from clinics that are randomized to PrEP and SRH provision only.

Missing data in terms of data management may occur because of nonresponse and study attrition. We will analyze differential attrition in relation to participants’ key demographic characteristics. Based on our prior research conducted in South Africa with AGYW who engage in sex risk behaviors, we estimate an average retention rate of 90% over the repeated follow-ups [19, 37, 62]. We will address missingness by including demographic covariates that will serve as proxies for dropout and by conducting a sensitivity analysis.

### Ethical approval

#### Full experimental protocol approval and amendments

The experimental phase of the study was approved by the South African Medical Association Research Ethics Committee (SAMAREC), which serves as the Institutional Review Board (IRB) of Record, in addition to the Tshwane District Health Research Committee and the Skills Development for Tshwane Municipal Clinics. Since receiving approval to conduct the full study, we amended the protocol to improve the study design and procedures. Table 3 summarizes these approvals.

**Table 3:** Summary of Protocol Approval and Amendments

Date of approval	Protocol and Amendments
1/2019	Initial submission to conduct the experimental phase of the study
3/2019	Request for SAMAREC to be the IRB of Record
3/2019	Revised research protocol and supporting materials to prepare for the full study phase
4/2019	Amended full study research activities to allow for the provision of PrEP
7/2019	Addition of participant follow-up consent documents and the 3-month follow-up questionnaire
10/2019	Minor revisions to study screener and eligibility criteria to ensure participants are not currently enrolled in another HIV project and to the study protocol regarding follow-up on pregnancy outcomes of participants who become pregnant during the course of the study
11/2019	Reduced the number of clinic staff follow-up visits from 3 to 2 appointments—specifically, 4- and 8-month follow-up appointments
2/2020	Minor modifications to study documents and protocol to prevent co-enrollment of participants involved in other HIV studies in the Tshwane area
3/2020	Modification to allow project staff to complete at-home data entry of de-identified data, and the submission of a staff agreement of confidentiality for at-home data entry because of the COVID-19 lockdown
4/2020	Minor modifications to clinician and support staff follow-up questionnaires and consenting procedures
4/2020	Notification to the IRB of resumption of limited face-to-face appointment with study participants during the COVID-19 lockdown
4/2020	Submission of the COVID-19 questionnaire to be administered via telephone during the COVID-19 lockdown

With COVID-19, there have been barriers and disruptions to PrEP initiation and dispensing, which may alter some of the expected study outcomes, study activities and data collection. We attempted to address these unprecedented circumstances (as noted in Table 3) without compromising the rigor of the research design and ensuring that participants were able to stay on PrEP. Some of the strategies included getting personal protective equipment, ensuring a travel nurse was able to dispense PrEP, and when possible, providing refills longer than 30 days. We will be monitoring these circumstances as they are changing daily.

#### Dissemination

Study findings will be disseminated to participants, health care professionals, and other stakeholders through our established YAB and CCB, program newsletter, journal articles, conference presentations and also other targeted dissemination channels.

## Discussion

This article describes the study protocol for a research study aimed at evaluating the impact of a stigma and discrimination reduction training program for clinic staff and the efficacy of a multilevel HIV prevention strategy that addresses young women’s empowerment to include substance use, sexual risk, and GBV; and access to and use of SRH and PrEP.

The UNAIDS Fast-Track Approach agenda, which drives the 95–95-95 goals for HIV prevention and treatment by 2030, includes ambitious targets for reducing new HIV infections and achieving zero discrimination, including in healthcare settings [1]. To meet this agenda, we must reduce the incidence of HIV among AGYW who are the least likely to have access to HIV prevention, testing, and treatment services but carry the greatest burden of HIV. Furthermore, although PrEP is safe and is a highly effective prevention method, especially among individuals at high risk of HIV acquisition, PrEP uptake and adherence remains low among certain high priority populations, particularly AGYW. This is the first study of its kind in South Africa to attempt to address structural S&D in public health clinics as a way to improve PrEP readiness, uptake, and adherence among this population and in turn reduce HIV burden.

The study comprises some notable innovations: (1) developing a partnership with the South African Department of Health to reduce S&D by clinic staff; (2) engaging the study’s YAB and the CCB in the adaptation of the YWHC (YAB) and the S&D (CCB) interventions, study marketing materials (YAB), S&D reduction training (YAB) as stakeholders to share their voices, and the regular meetings. This is an important step towards involving community members in the dissemination and implementation of HIV-prevention structural interventions. Support for structural changes may increase when the people whom the intervention is likely to affect participate in the process [63]; and (3) other contextual factors, such as GBV and substance use, and using an evidence-based, gender-focused approach are addressed. Additionally, partnering with the NDoH and the public health clinics is facilitating capacity building through the standardized PrEP provider training that will be required when PrEP provision is scaled up to a population level. Because this study is being conducted in collaboration with public health clinics, it may bring to light challenges that need to be addressed prior to integrating PrEP delivery into public health facilities, such as workload, the need for task shifting or sharing, and the need to enhance PrEP’s general acceptance in usual care settings.

Few interventions currently exist that address the multiple levels of the psychosocial and community barriers that prevent AGYW from accessing healthcare services. The adapted YWHC provides a more comprehensive HIV prevention toolkit with PrEP and reproductive health as next generation science. If found to be efficacious, the YWHC plus addressing the structural S&D may help reduce barriers to HIV prevention among a key population with the greatest incidence—offering more hope for an HIV-free generation of young women.

## Trial status

Recruiting.

## Supplementary information

**Additional file 1: Figure File 1**. CONSORT Diagram.

**Additional file 2: Supplementary File 1.** Clinician baseline survey.

**Additional file 3: Supplementary File 2.** Support staff baseline survey.

**Additional file 4: Supplementary File 3***.* AGYW baseline survey.

**Additional file 5: Supplementary File 4**. Estimated Detectable Differences in PrEP Uptake between AGYW in the PrEP/SRH + YWHC arm vs. PrEP/SRH only, controlling for intervention arm of clinic.

**Additional file 6: Supplementary File 5.** Spirit checklist.

**Additional file 7: Supplementary File 6.** Ethical approval proofs.

## Data Availability

The study is currently enrolling; therefore, data collection is ongoing. The datasets used and/or analyzed during the current study will be available from the corresponding principal investigator when outcomes and publications are complete.

## Abbreviations

ACASI: Audio computer-assisted self-interviewing
AE: Adverse event
AGYW: Adolescent girls and young women
ARV: Antiretroviral
CCB: Community Collaborative Board
DBS: Dried blood spots
DD: Difference-in-differences
DREAMS: Determined, Resilient, Empowered, AIDS-free, Mentored and Safe
DSMB: Data and Safety Monitoring Board
DSMP: Data and Safety Monitoring Plan
FTC: Emtricitabine
GBV: Gender-based violence
GEE: Generalized estimating equations
HAST: HIV/AIDS, STI, and TB
HBV: Hepatitis B virus
HPLC: High-performance liquid chromatography
HPP: Health Policy Project
IRB: Institutional Review Board
MDMA: 3,4-Methylenedioxymethamphetamine
MDR-TB: Multi-drug resistant tuberculosis
NDoH: National Department of Health
PID: Participant identification number
PrEP: Pre-exposure prophylaxis
PrEPARE: *PRevention, Empowering, and PRotEcting*
QMP: Quality Management Plan
RRBA: Revised Risk Behavior Assessment
SAE: Serious adverse event
SAMAREC: South African Medical Association Research Ethics Committee
S&D: Stigma and discrimination
SRH: Sexual and reproductive health
STI: Sexually transmitted infection
TB: Tuberculosis
TDF: Tenofovir disoproxil fumarate
TFV-DP: Tenofovir diphosphate
UNAIDS: Joint United Nations Programme on HIV/AIDS
WBOT: Ward-based outreach team
WHC: Women’s Health CoOp
YAB: Youth Advisory Board
YWHC: Young Women’s Health CoOp
