# Supplementary material for: The PrEPARE Pretoria Project: protocol for a cluster-randomized factorial-design trial to prevent HIV with PrEP among adolescent girls and young women in Tshwane, South Africa
Source: BMC Public Health. 2020 Sep 15;20:1403. doi: 10.1186/s12889-020-09458-y (PMC7490774; doi:10.1186/s12889-020-09458-y)
Supplement: Supplementary file 2 — Additional file 2: Supplementary File 1. Clinician baseline survey. [file 12889_2020_9458_MOESM2_ESM.pdf]

Hard Copy Questionnaire checked by:
Staff ID |__|__|__|
Initials_______Date_________

PrEPARE- PRETORIA
CLINICIAN QUESTIONNAIRE
(Pretoria, South Africa)

IDENTIFYING INFORMATION

UniqueID:... |__|__|__|__|__|__|__|
DATE: ....|__|__| / |__|__| / |__|__|__|__|
DD MM YYYY

CLINIC NAME____

READ *Before you begin, I'd like to remind you that all your answers will be kept private. You have the right to refuse to answer any question without having to explain why you did so, and if you do not remember something exactly, we would still like your best guess.*

Please let the staff know if you have any questions

NOTE: REMEMBER TO READ THE CONFIDENTIALITY NOTICE BELOW BEFORE PROCEEDING

CONFIDENTIALITY NOTICE

The data collected in this instrument are protected by privacy laws. Disclosure of information reported in this document without your prior consent is prohibited and violators may be subject to fines of $5,000 (± Rand 30,000) and/or further prosecutions.

Questions about the instrument and study can be directed to Dr. Wendee Wechsberg at + 1-919-541-6422 or via e-mail at wmw@rti.org. Acknowledgment of the scales and items are summarized in the project manual. This study is sponsored by the Eunice Kennedy Shriver National Institute of Child Health & Human Development (NICHD) grant no. R01HD094629.

SECTION 1: BACKGROUND INFORMATION

First, we would like to ask about your background.

1. AGE ....|__|__|

2. GENDER: [1]❑ FEMALE [2]❑ MALE

3. What is the highest level of school you completed?

   [2]❑ Primary School
   [3]❑ Secondary School
   [4]❑ TVET College
   [5]❑ Technical/University of Technology
   [6]❑ University
   [7]❑ Nursing College
   [9]❑ Other_______________________________

4. In addition to your formal training, have you ever received training (either formal or in workshops) in the following subjects? (Check all that apply.)

	Yes	No
a. HIV and STI infection prevention	[1]❑	[2]❑
If yes, how long was the training (# days?) ___________		
b. HIV stigma and discrimination	[1]❑	[2]❑
If yes, how long was the training (# days?) ___________		
c. Care for pregnant or sexually active youth.........................	[1]❑	[2]❑
If yes, how long was the training (# days?) ___________		
d. Abortion or Termination of Pregnancy (TOP) services	[1]❑	[2]❑
If yes, how long was the training (# days?) ___________		
e. Adolescent and Youth Friendly Services	[1]❑	[2]❑
If yes, how long was the training (# days?) ___________		
f. Pre-exposure Prophylaxis (PrEP) for HIV prevention.........	[1]❑	[2]❑
If yes, how long was the training (# days?) ___________		
g. Patients' informed consent, privacy, and confidentiality	[1]❑	[2]❑
If yes, how long was the training (# days?)___________		
h. Gender Based Violence..	[1]❑	[2]❑
If yes, how long was the training (# days?) ___________		

5. How many years have you been working at this facility?
   [1]❑ 1-2 years [2]❑3-5 years [3]❑ More than 5 years [4]❑ 0-1 years

6. What best describes your current job in this facility **(the job you spend the majority of your time on)**? (Please tick only one)

   1❑ Physician
   2❑ Professional Nurse
   3❑ Enrolled Nurse
   4❑ Community Health Nurse
   5❑ Nurse Assistant
   6❑ Pharmacist
   7❑ Pharmacist Assistant
   8❑ Counselor (HAST)
   9❑ Other ________________________________

7a. Have you ever worked in a department that specializes in offering services in HIV prevention, care and treatment?

   1❑Yes 2❑No

   7b. If yes, do you ***currently*** work in a department that specializes in offering services in HIV prevention?

   1❑Yes 2❑No

8. Have you ever worked in a department that provided sexual and reproductive health (SRH), family planning, condoms, STI or other services to young people (ages 16-24)?

   1❑Yes 2❑No

9. ***In the past three months***, approximately how many adolescent girls (ages 16-17) have you personally provided with sexual and reproductive health care or services?

   1❑ 0 2❑ 1-99 3❑100 -200 4❑ More than 200

10. ***In the past three months***, approximately how many young women (ages 18-24) have you personally provided with sexual and reproductive health care or services?

   1❑ 0 2❑ 1-99 3❑100 -200 4❑ More than 200

SECTION 2: PROVIDING SERVICES

Now I would like to ask you specifically about the services that you personally provide and that are provided at your facility

Q11a. What is the youngest age you believe a young girl should be allowed to receive birth control or family planning?

______Years

Q11b.What is the youngest age you believe a young girl should be allowed to receive HIV counseling and testing?

______Years

Q12a. What is the youngest age your facility allows a young girl to receive birth control or family planning?

______Years

Q12b. What is the youngest age your facility allows a young girl to receive HIV counseling and testing?

______Years

13. What do you do for adolescent girls (16-17) wanting sexual and reproductive health services? *(If you do not provide sexual and reproductive health services, select not-applicable)*

    a. Refer them to another provider or facility that can better care for them

    1❑Yes 2❑ No 3❑ Not applicable

    b. Strongly advise them against engaging in sexual activity

    1❑Yes 2❑ No 3❑ Not applicable

    c. Ask them to return with a parent or guardian so that we can provide them with services

    1❑Yes 2❑ No 3❑ Not applicable

    d. Have them wait until we have finished taking care of adult patients who have come for services.

    1❑Yes 2❑ No 3❑ Not applicable

14. What do you do for young women (18-24) who are not married wanting sexual and reproductive health services? *(If you do not provide sexual and reproductive health services, select not-applicable)*

    a. Refer them to another provider or facility that can better care for them

    1❑Yes 2❑ No 3❑ Not applicable

    b. Strongly advise them against engaging in sexual activity

    1❑Yes 2❑ No 3❑ Not applicable

15. How many times have you EVER prescribed HIV post-exposure prophylaxis (PEP) after unprotected sex or rape to adolescents and young women ages 16-24 years?

    1❑ Zero times
    2❑ Once
    3❑ 2-5 times
    4❑ 6-10 times
    5❑ More than 10 times

SECTION 3: KNOWLEDGE AND ATTITUDES TOWARD THE PROVISION OF PRE-EXPOSURE PROPHYLAXIS (PrEP)

Now we will ask you about your knowledge of PrEP, the provision of PrEP in your health facility, your concerns about PrEP and your perceptions of your colleagues' concerns.

16. Have you heard of PrEP?

1❑Yes 2❑No

IF YOU HAVE NOT HEARD OF PrEP, PLEASE MOVE TO QUESTION 24

17. Have you had any training on PrEP?

1❑Yes 2❑No

IF YOU HAVE NO TRAININGIN PrEP, PLEASE MOVE TO QUESTION 24

18. How long was the training?

1❑ Brochures or pamphlets only
2❑ 0-59 mins
3❑ 1 hour
4❑ 2-3 hours
5❑ 4-5 hours
6❑ 6 or more hours

Please indicate which answer is best.

19. According to current PrEP guidelines, is HIV testing required before initiating PrEP?

1❑Yes 2❑ No 3❑ I don't know

20. How many antiretroviral medications are in the regimen that is approved for PrEP?

1❑ 1
2❑ 2
3❑ 3
4❑ 4
5❑ I don't know

21. Which antiretroviral medication(s) are approved for PrEP? (Tick all that apply)

1❑ Zidovudine, also known as AZT or Retrovir
2❑ Lopinavir, also known as Kaletra
3❑ Lamivudine, also known as 3TC or Epivir
4❑ Efavirenz, also known as Sustiva
5❑ Emtricitabine + Tenofovir, also known as Truvada or Tenemine
6❑ Tenofovir, also known as Viread
7❑ Emtricitabine + Tenofovir + efavirenz, also known as Atripla
8❑ Emtricitabine, also known as FTC or Emtriva
9❑ I don't know

22. What is the approved dosing for the antiretrovirals used for PrEP?

- 1❑ Immediately prior to sexual activity
- 2❑ Immediately following sexual activity
- 3❑ Once daily
- 4❑ Twice daily
- 5❑ Three times per day
- 6❑ Once weekly
- 7❑ I don't know

23. How often should individuals be tested for HIV while taking PrEP?

- 1❑ Weekly
- 2❑ Monthly
- 3❑ Every 2-3 months
- 4❑ Every 6 months
- 5❑ Annually
- 9❑ Other: ______________________
- 10❑ I don't know

24.

DESCRIPTION OF PrEP

PrEP stands for Pre-Exposure Prophylaxis. PrEP is a pill that is taken every day to prevent HIV. PrEP is for people who do not have HIV but are worried about getting it and want to stay HIV negative. PrEP contains some of the medicines in ARVs. Research has shown that PrEP is safe. PrEP involves taking one tablet (swallowed by mouth) every day. It must be taken every day in order to be effective.

PrEP protects against HIV infection much like a malaria pill protects against malaria or a daily birth control pill protects against pregnancy. PrEP does not protect against other STIs or pregnancy and it is not a cure for HIV.

If I provide PrEP to adolescent girls (16-17) I am worried that:

a. Having access to PrEP will lead them to be reckless or take more sexual risks

1❑Not worried 2❑A little worried 3❑Worried 4❑Very worried 5❑Not applicable

b. I will be accused by the community of encouraging young women to have sex.

1❑Not worried 2❑A little worried 3❑Worried 4❑Very worried 5❑Not applicable

c. They might have difficulty in adhering to the daily PrEP regimen

1❑Not worried 2❑A little worried 3❑Worried 4❑Very worried 5❑Not applicable

d. My work colleagues will look down on me for providing PrEP to adolescent girls and young women because they think it will lead them to have risky behavior

[1]❑Not worried [2]❑A little worried [3]❑Worried [4]❑Very worried [5]❑Not applicable

e. Pregnancy rates among them will go up because they will stop using condoms if they are using PrEP.

[1]❑Not worried [2]❑A little worried [3]❑Worried [4]❑Very worried [5]❑Not applicable

f. Other sexually transmitted infections will increase because they will stop using condoms.

[1]❑Not worried [2]❑A little worried [3]❑Worried [4]❑Very worried [5]❑Not applicable

g. They will experience side effects from the PrEP and I will be blamed.

[1]❑Not worried [2]❑A little worried [3]❑Worried [4]❑Very worried [5]❑Not applicable

h. Their parents may become upset with me for giving them PrEP

[1]❑Not worried [2]❑A little worried [3]❑Worried [4]❑Very worried [5]❑Not applicable

25. What are some reasons that it would be difficult to dispense PrEP to adolescent girls or young women? ***(tick all options that apply)***

[1]❑ Not enough staff
[2]❑ Not enough time
[3]❑ Lack of training to provide PrEP
[4]❑ Lack of youth friendly services
[5]❑ Lack of enough PrEP stock
[6]❑ Patients will not adhere to their medication
[7]❑ Different language

SECTION 4: HEALTH FACILITY ENVIRONMENT

Now we will ask about practices/measures in your health facility and your experiences working in this facility.

26. In the past 3 months, how often have you observed clinic staff unwilling to care for:

a. Adolescent girl (16-17 years old) who is wanting any services in this facility?

[1]❑Never [2]❑Once or twice [3]❑Several times [4]❑Most of the time

b. Pregnant adolescent girl (16-17 years old) who is wanting antenatal care?

[1]❑Never [2]❑Once or twice [3]❑Several times [4]❑Most of the time

c. Adolescent girl (16-17 years old) who is wanting birth control services?

[1]❑Never [2]❑Once or twice [3]❑Several times [4]❑Most of the time

d. Adolescent girl (16-17 years old) who is wanting services for STI symptoms?

[1]❑Never [2]❑Once or twice [3]❑Several times [4]❑Most of the time

27. In the past 3 months, how often have you observed clinic staff talking badly about:

a. Adolescent girl (16-17 years old) who is wanting any services in this facility?

[1]❑Never [2]❑Once or twice [3]❑Several times [4]❑Most of the time

b. A patient living with HIV?

[1]❑Never [2]❑Once or twice [3]❑Several times [4]❑Most of the time

c. A pregnant adolescent girl (16-17 years old) who is wanting antenatal care?

[1]❑Never [2]❑Once or twice [3]❑Several times [4]❑Most of the time

d. Adolescent girl (16-17 years old) who is wanting birth control services?

[1]❑Never [2]❑Once or twice [3]❑Several times [4]❑Most of the time

e. Adolescent girl (16-17 years old) who is wanting services for STI symptoms?

[1]❑Never [2]❑Once or twice [3]❑Several times [4]❑Most of the time

28. In the past 3 months, how often have you observed clinic staff disclosing the health or sexual activity status of an adolescent girl (16-17) or young woman (18-24) without the young woman's permission, in a situation that was not medically required?

[1]❑Never [2]❑Once or twice [3]❑Several times [4]❑Most of the time

29. Please indicate if you agree or disagree with the following statements:

a. Some adolescent girls and young women (ages 16-24) come to this clinic because they know they will get confidential services

[1]❑Strongly Agree [2]❑Agree [3]❑Disagree [4]❑Strongly Disagree

b. Some adolescent girls and young women (ages 16-24) come to this clinic because they know providers will treat them with respect

[1]❑Strongly Agree [2]❑Agree [3]❑Disagree [4]❑Strongly Disagree

c. Some adolescent girls and young women (ages 16-24) do not come to the clinic because they know they will be shouted at, lectured and asked many questions

1❑Strongly Agree 2❑Agree 3❑Disagree 4❑Strongly Disagree

30. How hesitant are you to take an HIV test, in this facility, due to fear of other people's reactions if the test is positive?

1❑ Not hesitant 2❑ A little hesitant 3❑ Somewhat hesitant 4❑ Very hesitant

31. How sure are you that when you take an HIV test in this facility the results will be kept private?

1❑ Not sure 2❑ A little sure 3❑ Maybe sure 4❑ Very sure

SECTION 6: OPINIONS ABOUT PROVIDING SERVICES TO SEXUALLY ACTIVE YOUNG WOMEN

32. Do you strongly agree, agree, disagree, or strongly disagree with the following sentences?

a. Adolescent girls and young women will want PrEP because they are engaging in risky behaviour.

1❑Strongly Agree 2❑Agree 3❑Disagree 4❑Strongly Disagree

b. Adolescent girls and young women want birth control/family planning because they are engaging in risky behaviour.

1❑Strongly Agree 2❑Agree 3❑Disagree 4❑Strongly Disagree

c. Talking harshly to adolescent girls and young women wanting birth control/family planning is right because they are engaging in sexual behavior.

1❑Strongly Agree 2❑Agree 3❑Disagree 4❑Strongly Disagree

d. Talking harshly to adolescent girls and young women wanting PrEP is right because they are engaging in sexual behaviour.

1❑Strongly Agree 2❑Agree 3❑Disagree 4❑Strongly Disagree

e. It is important to strongly advise adolescent girls and young women who are wanting PrEP to stop having sex.

1❑Strongly Agree 2❑Agree 3❑Disagree 4❑Strongly Disagree

f. It is important to strongly advise adolescent girls and young women who are wanting birth control/family planning to stop having sex.

1❑Strongly Agree 2❑Agree 3❑Disagree 4❑Strongly Disagree

g. Providing PrEP to adolescent girls and young women will encourage them to have risky behaviour.

1❑Strongly Agree 2❑Agree 3❑Disagree 4❑Strongly Disagree

h. Providing birth control/family planning to adolescent girls and young women will encourage them to have risky behaviour.

1❑Strongly Agree 2❑Agree 3❑Disagree 4❑Strongly Disagree

i. PrEP should only be provided to adolescent girls under the age of 18 if their parent has accompanied them and provides consent.

1❑Strongly Agree 2❑Agree 3❑Disagree 4❑Strongly Disagree

j. If a young married woman wants birth control/family planning, her husband should be informed.

1❑Strongly Agree 2❑Agree 3❑Disagree 4❑Strongly Disagree

k. If a young married woman wants PrEP, her husband should be informed.

1❑Strongly Agree 2❑Agree 3❑Disagree 4❑Strongly Disagree

l. Adolescent girls and young women who want birth control/family planning are acting responsibly to look after their own health.

1❑Strongly Agree 2❑ Agree 3❑ Disagree 4❑Strongly Disagree

m. Adolescent girls and young women who want PrEP are acting responsibly to look after their own health.

1❑Strongly Agree 2❑ Agree 3❑ Disagree 4❑Strongly Disagree

33. Do you strongly agree, agree, disagree, or strongly disagree with the following statements?

a. I would treat the adolescent girl and young woman like my daughter if she were wanting sexual and reproductive health services

1❑ Strongly Agree
2❑ Agree
3❑ Disagree
4❑ Strongly Disagree

b. If I had a choice, I would prefer not to provide sexual and reproductive health services to sexually active adolescent girls ages 16-17

1❑ Strongly Agree
2❑ Agree
3❑ Disagree
4❑ Strongly Disagree

c. If I had a choice, I would prefer not to provide sexual and reproductive health services to unmarried sexually active young women ages 18-24

- 1☐ Strongly Agree
- 2☐ Agree
- 3☐ Disagree
- 4☐ Strongly Disagree
