# Supplementary material for: The PrEPARE Pretoria Project: protocol for a cluster-randomized factorial-design trial to prevent HIV with PrEP among adolescent girls and young women in Tshwane, South Africa
Source: BMC Public Health. 2020 Sep 15;20:1403. doi: 10.1186/s12889-020-09458-y (PMC7490774; doi:10.1186/s12889-020-09458-y)
Supplement: Supplementary file 3 — Additional file 3: Supplementary File 2. Support staff baseline survey. [file 12889_2020_9458_MOESM3_ESM.pdf]

Hard Copy Questionnaire checked by:
Staff ID |__|__|__|
Initials_______Date_________

PrEPARE- PRETORIA
SUPPORT STAFF QUESTIONNAIRE
(Pretoria, South Africa)

IDENTIFYING INFORMATION

Unique ID:... |__|__|__|__|__|__|__|
DATE: ....|__|__| / |__|__| / |__|__|__|__|
DD MM YYYY
CLINIC NAME____

READ *Before you begin, I'd like to remind you that all your answers will be kept private. You have the right to refuse to answer any question without having to explain why you did so, and if you do not remember something exactly, we would still like your best guess.*

Please let the staff know if you have any questions

NOTE: REMEMBER TO READ THE CONFIDENTIALITY NOTICE BELOW BEFORE PROCEEDING

CONFIDENTIALITY NOTICE

The data collected in this instrument are protected by privacy laws. Disclosure of information reported in this document without your prior consent is prohibited and violators may be subject to fines of $5,000 (± Rand 30,000) and/or further prosecutions.

Questions about the instrument and study can be directed to Dr. Wendee Wechsberg at + 1-919-541-6422 or via e-mail at wmw@rti.org. Acknowledgment of the scales and items are summarized in the project manual. This study is sponsored by the Eunice Kennedy Shriver National Institute of Child Health & Human Development (NICHD) grant no. R01HD094629.

SECTION 1: BACKGROUND INFORMATION

First, we would like to ask about your background.

1. AGE ....|__|__|

2. GENDER: FEMALE ¹❑ MALE ²❑

3. What is the highest level of school you completed?

   ¹❑ I have not had any school
   ²❑ Primary School
   ³❑ Secondary School
   ⁴❑ TVET College
   ⁵❑ Technical/University of Technology
   ⁶❑ University
   ⁷❑ Nursing College
   ⁹❑ Other___________________________

4. In addition to your formal training, have you ever received training (either formal or in workshops) in the following subjects? (Check all that apply.)

	Yes	No
a. HIV stigma and discrimination	1❑	2❑
If yes, how long was the training (# days?) _____________		
b. Providing services for youth ages 16-24 years old............	1❑	2❑
If yes, how long was the training (# days?) _____________		
c. Patients' informed consent, privacy, and confidentiality	1❑	2❑
If yes, how long was the training (# days?) _____________		

5. How many years have you been working at this facility?

   1❑ 1-2 years 2❑3-5 years 3❑ More than 5 years 4❑ 0-1 years

6. What best describes your current job in this facility **(the job you spend the majority of your time on)**? (Please tick only one)

   1❑ Administrative Clerk
   2❑ Clerk (other)
   3❑ Cashier
   4❑ Secretary
   5❑ Receptionist
   6❑ Accountant
   7❑ Cleaner
   8❑ Security
   9❑ Other ____

7a. Have you ever worked in a department that specializes in offering services in HIV prevention, care and treatment?

[1]❑Yes [2]❑ No

7b. If yes, do you ***currently*** work in a department that specializes in offering services in HIV prevention?

[1]❑Yes [2]❑ No

8. Have you ever worked in a department that provided sexual and reproductive health (SRH), family planning, condoms, STI or other services to young people (ages 16-24)?

[1]❑Yes [2]❑ No

SECTION 2: PROVIDING SERVICES

Now I would like to ask you specifically about the services that you personally provide and that are provided at your facility

Q9a. What is the youngest age you believe a young girl should be allowed to receive birth control or family planning?

______Years

Q9b. What is the youngest age you believe a young girl should be allowed to receive HIV counseling and testing?

______Years

Q10a. What is the youngest age your facility allows a young girl to receive birth control or family planning?

______Years

Q10b. What is the youngest age your facility allows a young girl to receive HIV counseling and testing?

______Years

11. What happens in this facility when teenage girls (16-17) want services such as receiving birth control and pregnancy care?

    a. They are referred to another facility that can better care for them.

    1❑ Yes 2❑ No 3❑ I don't know

    b. They are strongly advised against engaging in sexual activity.

    1❑ Yes 2❑ No 3❑ I don't know

    c. They are asked to return with a parent or guardian so that the facility can provide them with the services.

    1❑ Yes 2❑ No 3❑ I don't know

    d. They must wait until after all the adult patients who have come for services have been taken care of.

    1❑ Yes 2❑ No 3❑ I don't know

12. What happens in this facility when young unmarried women ages 18 -24 want birth control and pregnancy care services?

    a. They are referred to another facility that can better care for them.

    1❑ Yes 2❑ No 3❑ I don't know

    b. They are strongly advised against engaging in sexual activity.

    1❑ Yes 2❑ No 3❑ I don't know

SECTION 3: KNOWLEDGE AND ATTITUDES TOWARD THE PROVISION OF PRE-EXPOSURE PROPHYLAXIS (PrEP)

Now we will ask you about your knowledge of PrEP, the provision of PrEP in your health facility, your concerns about PrEP and your perceptions of your colleagues' concerns.

13. Have you heard of PrEP?

    1❑ Yes 2❑ No

<u>DESCRIPTION OF PREP</u>
PrEP stands for Pre-Exposure Prophylaxis. PrEP is a pill that is taken every day to prevent HIV. PrEP is for people who do not have HIV but are worried about getting it and want to stay HIV negative. PrEP contains some of the medicines in ARVs. Research has shown that PrEP is safe. PrEP involves taking one tablet (swallowed by mouth) every day. It must be taken every day in order to be effective.

PrEP protects against HIV infection much like a malaria pill protects against malaria or a daily birth control pill protects against pregnancy. PrEP does not protect against other STIs or pregnancy and it is not a cure for HIV.

14. If PrEP is provided to teenage girls (16-17) at this facility, I am worried that:

a. Having access to PrEP will lead them to be reckless or take more sexual risks.

1❑Not worried 2❑A little worried 3❑Worried 4❑Very worried

b. This facility will be accused by the community of encouraging young women to have sex.

1❑Not worried 2❑A little worried 3❑Worried 4❑Very worried

c. They might have difficulty in taking a PrEP pill every day.

1❑Not worried 2❑A little worried 3❑Worried 4❑Very worried

d. The number of pregnancies among them will go up because they will stop using condoms if they are using PrEP.

1❑Not worried 2❑A little worried 3❑Worried 4❑Very worried

e. Other sexually transmitted infections will increase because they will stop using condoms.

1❑Not worried 2❑A little worried 3❑Worried 4❑Very worried

f. They will experience side effects from the PrEP and the facility will be blamed.

1❑Not worried 2❑A little worried 3❑Worried 4❑Very worried

g. Their parents may become upset with the facility for giving them PrEP if they don’t know that their daughter is taking PrEP.

1❑Not worried 2❑A little worried 3❑Worried 4❑Very worried

SECTION 4: HEALTH FACILITY ENVIRONMENT

Now we will ask about practices/measures in your health facility and your experiences working in this facility.

15. In the past 3 months, how often have you observed clinic staff unwilling to care for:

a. Teenage girl who is wanting any services in this facility?

1❑Never 2❑Once or twice 3❑Several times 4❑Most of the time

b. Pregnant teenage girl who is wanting pregnancy care?

1❑Never 2❑Once or twice 3❑Several times 4❑Most of the time

c. Teenage girl who is wanting birth control services?

1❑Never 2❑Once or twice 3❑Several times 4❑Most of the time

d. Teenage girl who is wanting services for STI treatment?

1❑Never 2❑Once or twice 3❑Several times 4❑Most of the time

16. In the past 3 months, how often have you observed clinic staff talking badly about:

a. Teenage girl who is wanting any services in this facility?

1❑Never 2❑Once or twice 3❑Several times 4❑Most of the time

b. A patient living with HIV?

1❑Never 2❑Once or twice 3❑Several times 4❑Most of the time

c. A pregnant teenage girl who is wanting pregnancy care?

1❑Never 2❑Once or twice 3❑Several times 4❑Most of the time

d. Teenage girl who is wanting birth control services?

1❑Never 2❑Once or twice 3❑Several times 4❑Most of the time

e. Teenage girl who is wanting services for STI treatment?

1❑Never 2❑Once or twice 3❑Several times 4❑Most of the time

17. In the past 3 months, how often have you observed clinic staff disclosing the health or sexual activity of a teenage girl or young woman when it was not medically required?

    1❑Never 2❑Once or twice 3❑Several times 4❑Most of the time

18. Please indicate if you agree or disagree with the following statements:

    a. Some teenage girls and young women come to this clinic because they know they will get confidential services

       1❑ Strongly Agree 2❑ Agree 3❑ Disagree 4❑ Strongly Disagree

    b. Some teenage girls and young women come to this clinic because they know doctors and nurses will treat them with respect

       1❑ Strongly Agree 2❑ Agree 3❑ Disagree 4❑ Strongly Disagree

    c. Some teenage girls and young women do not come to the clinic because they know they will be shouted at, lectured and asked too many questions

       1❑ Strongly Agree 2❑ Agree 3❑ Disagree 4❑ Strongly Disagree

19. How hesitant are you to take an HIV test, in this facility, due to fear of other people's reactions if the test is positive?

    1❑ Not hesitant 2❑ A little hesitant 3❑ Somewhat hesitant 4❑ Very hesitant

20. How sure are you that when you take an HIV test in this facility the results will be kept private?

    1❑ Not sure 2❑ A little sure 3❑ Maybe sure 4❑ Very sure

SECTION 6: OPINIONS ABOUT PROVIDING SERVICES TO SEXUALLY ACTIVE YOUNG WOMEN

21. Do you strongly agree, agree, disagree, or strongly disagree with the following sentences?

    a. Teenage girls and young women will want PrEP because they are engaging in risky behaviour.

       1❑ Strongly Agree 2❑ Agree 3❑ Disagree 4❑ Strongly Disagree

    b. Teenage girls and young women want birth control/family planning because they are engaging in risky behaviour.

       1❑ Strongly Agree 2❑ Agree 3❑ Disagree 4❑ Strongly Disagree

    c. Talking harshly to teenage girls and young women wanting birth control/family planning is right because they are having sex.

       1❑ Strongly Agree 2❑ Agree 3❑ Disagree 4❑ Strongly Disagree

d. Talking harshly to teenage girls and young women wanting PrEP is right because they are having sex.

[1]❑ Strongly Agree [2]❑ Agree [3]❑ Disagree [4]❑ Strongly Disagree

e. It is important to strongly advise teenage girls and young women who are wanting PrEP to stop having sex.

[1]❑ Strongly Agree [2]❑ Agree [3]❑ Disagree [4]❑ Strongly Disagree

f. It is important to strongly advise teenage girls and young women who are wanting birth control/family planning to stop having sex.

[1]❑ Strongly Agree [2]❑ Agree [3]❑ Disagree [4]❑ Strongly Disagree

g. Providing PrEP to teenage girls and young women will encourage them to engage in risky behaviour.

[1]❑ Strongly Agree [2]❑ Agree [3]❑ Disagree [4]❑ Strongly Disagree

h. Providing birth control/family planning to teenage girls and young women will encourage them to engage in risky behaviour.

[1]❑ Strongly Agree [2]❑ Agree [3]❑ Disagree [4]❑ Strongly Disagree

i. PrEP should only be provided to teenage girls under the age of 18 if their parent has accompanied them and provides consent.

[1]❑ Strongly Agree [2]❑ Agree [3]❑ Disagree [4]❑ Strongly Disagree

j. If a young married woman wants birth control/family planning, her husband should be informed.

[1]❑ Strongly Agree [2]❑ Agree [3]❑ Disagree [4]❑ Strongly Disagree

k. If a young married woman wants PrEP, her husband should be informed.

[1]❑ Strongly Agree [2]❑ Agree [3]❑ Disagree [4]❑ Strongly Disagree

l. Teenage girls and young women who want birth control/family planning are acting responsibly to look after their own health.

[1]❑ Strongly Agree [2]❑ Agree [3]❑ Disagree [4]❑ Strongly Disagree

m. Adolescent girls and young women who want PrEP are acting responsibly to look after their own health.

[1]❑ Strongly Agree [2]❑ Agree [3]❑ Disagree [4]❑ Strongly Disagree
