# Supplementary material for: The PrEPARE Pretoria Project: protocol for a cluster-randomized factorial-design trial to prevent HIV with PrEP among adolescent girls and young women in Tshwane, South Africa
Source: BMC Public Health. 2020 Sep 15;20:1403. doi: 10.1186/s12889-020-09458-y (PMC7490774; doi:10.1186/s12889-020-09458-y)
Supplement: Supplementary file 4 — Additional file 4: Supplementary File 3. AGYW baseline survey. [file 12889_2020_9458_MOESM4_ESM.pdf]

Hard Copy Questionnaire checked by:
Staff ID |__|__|__| Initials_______Date_________

BASELINE

PRETORIA AGYW HEALTH COOP QUESTIONNAIRE

(Pretoria, South Africa)

IDENTIFYING INFORMATION

PARTICIPANT ID:..|__|__|__|__|__|__|__|

DATE: ....|__|__| / |__|__| / |__|__|__|__|
DD MM YYYY

STAFF ID:..|__|__|__|

START TIME:..|__|__| : |__|__|
(Use 24 hr clock)

Before we begin, we would like to remind you that all your answers will be kept private. You have the right to refuse to answer any question without having to explain why you did so, and if you do not remember something exactly, we would still like your best guess.

Please let the staff know if you have any questions.

NOTE: REMEMBER TO READ THE CONFIDENTIALITY NOTICE BELOW BEFORE PROCEEDING

CONFIDENTIALITY NOTICE

The data collected in this instrument are protected by privacy laws. Disclosure of information reported in this document without your prior consent is prohibited and violators may be subject to fines of $5,000 (± Rand 30,000) and/or further prosecutions.

Questions about the instrument and study can be directed to Dr. Wendee Wechsberg at + 1-919-541-6422 or via e-mail at wmw@rti.org. Acknowledgment of the scales and items are summarized in the project manual. This study is sponsored by the Eunice Kennedy Shriver National Institute of Child Health & Human Development (NICHD) grant no. R01HD094629.

Table of Contents

SECTION A: DEMOGRAPHICS 3
SECTION B: PRE-EXPOSURE PROPHYLAXIS (PREP) 7
SECTION C: ACCESS TO CARE & SERVICES 11
SECTION D: ALCOHOL & DRUG USE 13
SECTION E: NEED FOR ALCOHOL AND/OR DRUG TREATMENT 17
SECTION F: DEPRESSION & ANXIETY 18
SECTION G: POST TRAUMATIC STRESS 19
SECTION H: SEXUAL HISTORY 20
SECTION I: TRANSACTIONAL SEX 24
SECTION J: CONDOM NEGOTIATION & SEXUAL DISCUSSION 27
SECTION K: POWER AND EMPOWERMENT 28
SECTION L: ENVIRONMENT 29
SECTION M: HIV TESTING AND RISK 31
SECTION N: WOMEN’S HEALTH 32
SECTION O: LAST SEXUAL EPISODE 35
SECTION P: VICTIMISATION 37
SECTION Q: SOCIAL SUPPORT & AGENCY 43
SECTION R: HOPE FOR THE FUTURE 45

SECTION A: DEMOGRAPHICS

NOTE: UNLESS OTHERWISE SPECIFIED, SELECT ONLY ONE ANSWER PER QUESTION.

We would like to know a few things about you. Some of the questions are sensitive in nature and we want to assure you that your responses will be kept private. We understand that some of these are difficult topics, but please answer as much as you can.

A1. ***(PRBA)*** How old are you now?
AGE .... |__|__| [14-28]

A2. When were you born? If you don't know your exact birth date, please give your best guess.
YEAR ....|__|__|__|__|
DAY... |__|__|
MONTH.. |__|__|

CHECK: Verify consistency using A1, A2, and entered Date.

If A1 is 16-24 then goto A3a; else ask:

A1Cn. Just to confirm, how old are you? |__|__|......[14-28]

If A1Cn = A1 then goto InelA1; else ask A2Cn

A2Cn. Just to confirm, when were you born? If you don't know your exact birth date, please give your best guess.

CHECK: Verify consistency using A1Cn, A2Cn, and entered Date.

If A1Cn is 16-24 then goto A3a; else ask InelA1:

InelA1. Please alert a staff member for assistance. ___________
Staff must enter password ("prepare", not case sensitive) to end interview as ineligible.

A3a. ***(CT_TEEN)*** Have you ever left school before finishing Grade 12 (Matric)?

YES .... ❑1
NO... ❑2

IF A3a=NO, DK, RF, SKIP TO A3c

A3b. ***(CT_TEEN)*** Have you ever gone back to school?

YES .... ❑1
NO... ❑2

A3c. ***(PRBA)*** What is the highest standard/grade that you completed at school?
Less than one year completed..0
Sub A/Class 1/Grade 1..1
Sub B/Class 2/Grade 2..2
Standard 1/Grade 3..3
Standard 2/Grade 4..4
Standard 3/Grade 5..5
Standard 4/Grade 6..6
Standard 5/Grade 7..7
Standard 6/Grade 8..8
Standard 7/Grade 9..9
Standard 8/Grade 10..10
Standard 9/Grade 11..11
Standard 10/Grade 12..12
Passed matric..13
Got a matric exemption (ABET)..14

A4a. ***(NEW)*** What other education have you completed?
(CHECK ALL THAT APPLY)
Apprenticeship..1
Certificate program..2
Diploma/ trade or technical training..3
Short courses (for example secretarial)..4
College (or university) degree..5
None..0
(DO NOT ALLOW “NONE” (0) WITH ANY OTHER OPTION)

A4b. ***(CT_TEEN)*** Do you want to study further?
YES..❑1
NO……………………………………………………………...❑2

A4c. ***(CT_TEEN)*** Are you going to school now?
YES..❑1
NO..❑2

A5. ***(PRBA-MOD)*** What is your current relationship status? Please choose the best response for your current relationship.
Single (no boyfriend, girlfriend, or husband)..1
Have a boyfriend or girlfriend not living together..2
Have a boyfriend or girlfriend living together..3
Married..4
Separated..5
Divorced..6
Widowed..7

A6a. ***(RRBA - MOD)*** Where are you living or staying now?
(CHECK ALL THAT APPLY)
In a home or flat (owned by you, your parents, or someone else)..... ❑1
In an informal settlement or shacks .... ❑2
In an abandoned building, empty lot .... ❑3
On the streets/park .... ❑4
In a rented room/space.. ❑5
In a shelter .... ❑6
In a backyard dwelling (wendy house) .... ❑7
Dorm/boarding school .... ❑8
In some other place .... ❑99
Specify the other place you are staying____________________________

A6b. ***(PRBA - MOD)*** Where you live now, do you have running water inside the building?
YES 1
NO...2

A6c. ***(RRBA - MOD)*** Do you consider yourself homeless now (that is, you have nowhere to stay)?
YES 1
NO...2

A7. ***(PRBA)*** Who do you live with now?
(CHECK ALL THAT APPLY)
Parent/Guardian(s) .... ❑1
My Child/children.. ❑2
Other Family (including siblings).......................... ❑3
My male partner .... ❑4
Male partners' family………………………… .... ❑5
Friends .... ❑6
Blessor (Sugar Daddy) .... ❑7
Gang Members .... ❑8
Drug Dealer/Merchant .... ❑9
Someone else .... ❑99
Specify who else you live with __.____
(DO NOT ALLOW "ALONE" (0) WITH ANY OTHER OPTION)
Alone .... ❑0

A8a. ***(WHC+ - NIDA STTR)*** During the past 3 months (90 days), how often did your household (people you live with) run out of money for basic necessities like food?
Never.. ❑0
Less than monthly .... ❑1
Monthly... ❑2
Weekly .... ❑3
Every day or almost every day.................... ❑4

A8b. ***(WHC+)*** How often in the past 3 months did you go to bed hungry?

Never.. ❑0
Less than monthly .... ❑1
Monthly... ❑2
Weekly .... ❑3
Every day or almost every day .... ❑4

Please answer the next question about your employment or work.

A10. ***(PRBA - MOD)*** Which most closely describes your status?

(CHECK ALL THAT APPLY)

Employed full-time.. ❑1
Employed part-time .... ❑2
Self-employed full-time .... ❑3
Self-employed part-time .... ❑4
Retrenched (laid off)... ❑5

(DO NOT ALLOW "UNEMPLOYED" [6,7] WITH EMPLOYED OPTIONS [1, 2, 3, 4])

Unemployed, looking for job .... ❑6
Unemployed, not looking for job .... ❑7

(DO NOT ALLOW BOTH OPTIONS 6 AND 7 TOGETHER)

Student (secondary) .... ❑8
Student (college) .... ❑9
Disabled .... ❑10
OTHER ❑99

SECTION B: PRE-EXPOSURE PROPHYLAXIS (PREP)

We would like to know about your awareness of PrEP, a medication to prevent HIV.

B1. ***(NC CLINICS)*** Before you enrolled in this study, had you heard of PrEP, a daily pill that a person who does *not* have HIV can take to help them stay HIV negative (for example, before having sex with someone who is HIV-positive)?
YES .... ❑1
NO .... ❑2 **(GO TO B3)**
DON'T KNOW .... ❑-7 **(GO TO B3)**

**B2.** ***(HPTN 082 [MOD] – Ali M)*** Please check YES or NO from what you know about PrEP, pills that prevent HIV.	Yes	No
**PrEP Knowledge**		
a) PrEP is a pill that needs to be taken daily to protect against HIV.	❑1	❑2
b) PrEP is safe for adolescents.	❑1	❑2
c) PrEP protects against getting other sexually transmitted infections.	❑1	❑2
d) PrEP protects against falling pregnant.	❑1	❑2

B3. ***(NC CLINICS)*** Have you ever TAKEN a pill to prevent HIV (PrEP or PEP)?
YES... 1
NO.. 2 **(GO TO B4)**

B3a. ***(NEW)*** When you took a pill to prevent HIV, was it PrEP or PEP? Meaning, did you take the pill before being exposed to someone who might have HIV (PrEP) or after you were exposed to someone who might have HIV (PEP).
PrEP(before you might have been exposed to HIV)...................... 1
PEP (after you might have been exposed to HIV)......................... 2
PrEP and PEP.. 3

PrEP stands for pre-exposure prophylaxis. PrEP is a daily pill that can be given to HIV-negative individuals to prevent them from becoming infected with HIV. People taking PrEP will need to take an HIV test before they start and again every 3 months. If people take PrEP at the same time every day, the medication can be over 90% effective in preventing them from getting HIV. However, PrEP does not prevent sexually transmitted infections (STIs) or pregnancy.

B4. ***(NC CLINICS)*** How likely would you be to take PrEP (daily HIV prevention pill) if it were available for free?
Definitely would not take PrEP 1
Probably would not take PrEP... 2
Might take PrEP 3
Probably would take PrEP... 4
Definitely would take PrEP 5

B5a. ***(NEW)*** Would you be comfortable going to a clinic that has youth friendly services (services for teens or young people) to get PrEP?
YES... 1
NO.. 2

B5b. ***(NEW)*** Would you be comfortable going to a clinic that is NOT youth friendly to get PrEP?

YES..1
NO..2

B6. ***(NC CLINICS)*** If you began taking PrEP, do you think it would affect how often you use condoms?

I would be MORE likely to use condoms with partner(s) if I was taking PrEP 1
Condom use would not change if I was taking PrEP.....................2
I would be LESS likely to use condoms if I was taking PrEP.........3

B8. ***(HPTN 082 - MOD)*** Please let us know how strongly you AGREE or DISAGREE with the following statements about PrEP.

	Strongly Disagree	Disagree	Agree	Strongly Agree	N/A
**PrEP Readiness**					
a) I am ready to start taking PrEP to protect against HIV.	❑1	❑2	❑3	❑4	
b) I know the benefits of taking PrEP.	❑1	❑2	❑3	❑4	
c) I know that I will be able to take my PrEP daily.	❑1	❑2	❑3	❑4	
**PrEP Disclosure**					
d) If the statement doesn't apply to you please select N/A. For example, if you don't live with anyone, select N/A as your response.<br><br>I would tell most of the people that I live with if I was taking PrEP.	❑1	❑2	❑3	❑4	❑5
e) I would tell most of my friends if I was taking PrEP.	❑1	❑2	❑3	❑4	❑5
f) Please Note: A main male partner is a boyfriend, bae, or husband. A main male partner is also called your 'main man,' 'straight partner' or ngqo. If you don't have a main male partner, select N/A/<br><br>I would tell my main male partner (*boyfriend, bae, husband*) if I was taking PrEP	❑1	❑2	❑3	❑4	❑5
g) I would tell other sexual partners if I was taking PrEP	❑1	❑2	❑3	❑4	❑5
**PrEP-Related Social Support**					
h) I think my household members who I would tell that I am on PrEP would help me remember to take it.	❑1	❑2	❑3	❑4	❑5
i) I think my friends who I would tell I am on PrEP would help me remember to take it.	❑1	❑2	❑3	❑4	❑5
j) I think my main male partner would support me if I took PrEP.	❑1	❑2	❑3	❑4	❑5
**Reaction to Disclosure**					

k) My main male partner would be violent toward me if I told him that I was taking PrEP.	❑1	❑2	❑3	❑4	❑5
l) My main male partner would accuse me of having other partners if I told him I was taking PrEP	❑1	❑2	❑3	❑4	❑5
m) My family would shame me if I told them I was taking PrEP	❑1	❑2	❑3	❑4	❑5
**Perceived Efficacy**					
n) I believe taking PrEP can keep me from getting HIV.	❑1	❑2	❑3	❑4	
**Health Seeking Self- Efficacy**					
o) I would know how to contact the health clinic if I had problems or questions about PrEP.	❑1	❑2	❑3	❑4	
p) I would know who to call and where to go for refills of my PrEP.	❑1	❑2	❑3	❑4	
**Barriers to PrEP Uptake and Adherence**					
q) I have a private place to keep PrEP.	❑1	❑2	❑3	❑4	
r) I would take PrEP even if I was drinking alcohol.	❑1	❑2	❑3	❑4	
s) I would take PrEP even if I was doing drugs.	❑1	❑2	❑3	❑4	
t) I think that taking PrEP would give me side effects.	❑1	❑2	❑3	❑4	

Please answer the following questions about your thoughts about possible PrEP use.

B11. ***(HPTN 082 - MOD)*** Please tell us how much you agree or disagree with the following statements about what would happen if you were taking PrEP:

	Strongly Disagree	Disagree	Agree	Strongly Agree
a) I would feel ashamed of using PrEP.	❑1	❑2	❑3	❑4
b) ***(NEW)*** People would think I was loose if I was taking PrEP.	❑1	❑2	❑3	❑4

People may miss taking their medications for various reasons. If you were taking PrEP, what might keep you from taking it every day?

B14. ***(WHC+ - MOD FOR PrEP)***

I might miss taking the PrEP pill every day because I…	**Yes**	**No**
a. Want to avoid side effects (like upset stomach).	☐ 1	☐ 2
b. Would not have food.	☐ 1	☐ 2
c. Would not fully understand how to take PrEP.	☐ 1	☐ 2
e. Would not have transportation to go pick up my refill at the clinic or pharmacy.	☐ 1	☐ 2
f. Would lose the pills.	☐ 1	☐ 2

g. Would forget.	☐ 1	☐ 2
i. Would get tired of taking the pill every day.	☐ 1	☐ 2
j. Would fear that someone would find my bottle of PrEP pills	☐ 1	☐ 2
l. Because of other reasons? *Specify:* ________________________________	☐ 1	☐ 2

If PrEP was available in your clinic, young women interested in taking PrEP would need to be tested to make sure they are not HIV positive, are not pregnant, and have a healthy liver.

B15a. ***(NEW)*** When would you be ready to start taking PrEP, if it was available in your clinic?

I have not thought about it.. 1
I am not ready... 2
I am ready to start in the next 6 months... 3
I am ready to start in the next 3 months... 4
I am ready to start in the next month.. 5

SECTION C: ACCESS TO CARE & SERVICES

We would like to know a little about your access and use of healthcare services. Please tell us about your ability to get health care during the past 3 months (90 days).

C1. ***(NEW)*** Do you know where to go to get clinic services when you need them?

YES ....❑1
NO..❑2

C2. ***(NEW)*** How often, in your lifetime (ever) have you had a problem using clinic services when you needed them?

Never ....❑1
Rarely..❑2
Sometimes ....❑3
Often ....❑4
Almost Always...❑5

C3. ***(NEW – Based on Formative)*** I am going to read some reasons people may not be able to go to clinics.

You have not been able to get health care because…	Ever		Did this happen in the past 3 months?	
	**Yes**	**No**	**Yes**	**No**
a) You did not have transport	❑1	❑2 (GO TO C3b)	❑1	❑2
b) You did not understand the forms at the clinic	❑1	❑2 (GO TO C3c)	❑1	❑2
c) The nurses and clerks were harsh	❑1	❑2 (GO TO C3d)	❑1	❑2
d) You did not think that the nurses would help you with your problems	❑1	❑2 (GO TO C3e)	❑1	❑2
e) The nurses were not friendly to young women like you	❑1	❑2 (GO TO C3f)	❑1	❑2
f) You were scared that the clinic staff would share your private information	❑1	❑2 (GO TO C3g)	❑1	❑2
g) You were afraid that others in the community would see you	❑1	❑2 (GO TO C3h)	❑1	❑2
h) You were afraid that people would spread rumors about why you went to the clinic	❑1	❑2 (GO TO C8)	❑1	❑2

Please answer a few questions about your experience with clinic staff (e.g. doctors, nurses, health counsellors). Please remember that this information is private.

C8. ***(NEW – Based on Formative)*** Have you had any of the following experiences with staff at the clinics?

Please respond Yes or No ***based on your experience with clinic staff at the clinics.***	**Yes**	**No**	**Did this happen in the past 3 months? Yes/No**	
a) The clinic staff said harsh things because I asked for birth control.	❑1	❑2 (GO TO C8b)	❑1	❑2
b) The clinic staff said harsh things because I asked for an HIV test.	❑1	❑2 (GO TO C8c)	❑1	❑2

c) The clinic staff treated me badly because of my age.	❑1	❑2 (GO TO C8d)	❑1	❑2
d) The clinic staff told my family that I went to the clinic.	❑1	❑2 (GO TO C8e)	❑1	❑2
e) The clinic staff told other people in the community that I went to the clinic.	❑1	❑2 (GO TO C8f)	❑1	❑2
f) The clinic staff gossiped about me.	❑1	❑2 (GO TO C8g)	❑1	❑2
g) The clinic staff looked down on me because of how I looked.	❑1	❑2 (GO TO C8h)	❑1	❑2
h) The clinic staff looked down on me because of the community that I live in.	❑1	❑2 (GO TO C8i)	❑1	❑2
i) Is there another way you felt judged or shamed by clinic staff? Specify__________	❑1	❑2 (GO TO C5)	❑1	❑2

C5. ***(NEW)*** Have you ever not gone for health care because you were afraid that clinic staff may judge or shame you?

YES.. 1
NO.. 2

C4. ***(NEW – based on WHC+)*** Have you ever been judged or shamed by clinic staff?

YES.. 1
NO.. 2

LOGIC: IF ANY OF C8a-I = YES OR C4 = YES THEN ASK C6, ELSE SKIP TO C9

C6. ***(NEW)*** Have you ever stopped going for services at a healthcare clinic because you were judged or shamed?

YES.. 1
NO.. 2

C7a. ***(NEW)*** In the past 3 months, have you been judged or shamed by clinic staff?

YES.. 1
NO.. 2

C7b. ***(NEW)*** In the past 3 months, have you stopped going for services at a healthcare clinic because you were judged or shamed?

YES.. 1
NO.. 2

C9. Have you ever been refused (not been given) services by clinic staff?

YES.. 1
NO.. 2

SECTION D: ALCOHOL & DRUG USE

We are now going to ask about your alcohol drinking behavior. Please note that one drink is equivalent to one can or bottle of beer, cider or coolers, one glass of wine, or one tot of spirits.

D1. **(YWC)** When was the last time you drank alcohol?

Within the past 2 days .... ❑1
3-6 days ago............ ❑2
1-3 weeks ago .... ❑3
1-3 months ago............ ❑4
4-12 months ago............ ❑5 **(GO TO D6)**
More than 12 months ago............ ❑6 **(GO TO D6)**
Never............ ❑7 **(GO TO D6)**

Please answer these questions about your alcohol use during the past 3 months (90 days).

D2. **(Modified – AUDIT)** How many days in the past 3 months have you drank any kind of alcohol?
(**PROBE:** What is your best guess?)
OF DAYS .... |__|__| [1-90]

D3. **(Modified – AUDIT)** On an average day of drinking about how many drinks did you have, in the past 3 months? What is your best guess?
OF DRINKS............ |__|__| [1-99]

D4. **(Modified – GAIN I 1999)** How many days in the past 3 months did you drink four or more drinks of alcohol at one time? What is your best guess?
OF DAYS .... |__|__| [CHECK: D4 ≤ D2] [0-90]

D4a. **(Modified – Couples)** Which days of the week do you typically drink?

(CHECK ALL THAT APPLY)
Monday 1
Tuesday 2
Wednesday 3
Thursday 4
Friday 5
Saturday............ 6
Sunday............ 7

D5. **(Brief YAACQ - MOD)** Following is a list of things that sometimes happen either during or after drinking alcohol.

Please let us know if any of these have happened to you in the past 3 months.	**YES** ▼	**NO** ▼
a. I have taken foolish risks when I have been drinking.	1	2
b. I have gotten into physical fights because of drinking.	1	2
c. I have damaged property, or done something disruptive such as setting off a false fire alarm, or other things like that after I had been drinking.	1	2
d. As a result of drinking, I neglected to protect myself and condomize (use a condom).	1	2

e. When drinking, I have done impulsive (foolish) things that I regretted later.	1	2
f. My drinking has gotten me into sexual situations I later regretted.	1	2
g. I have injured someone else while drinking or drunk.	1	2
h. I have injured myself while drinking or drunk.	1	2

Now we are going to ask you some questions about drugs that you may have used in the past or still use.

D6. (Modified - RBA) Have you ever used any drugs (including dagga)?
YES... 1
NO.. 2 **(GO TO D16)**

D7x: (Modified – RBA) How many **days** have you used _____ in the last 3 months? What is your best guess?

a) Marijuana (dagga/cannabis/paji/zolo/weed/ ganja/ntsango)	|__|__|
b) Tik (Methamphetamine)	|__|__|
c) Cocaine (coke, stone, rock) that you injected or snorted	|__|__|
d) Heroin	|__|__|
e) Nyaope/Thinka	|__|__|
f) Mandrax (buttons, white pipe, pille)	|__|__|
g) Inhalants (glue, petrol)	|__|__|
h) Ecstasy (E), Club Drugs	|__|__|
i) Other amphetamines or uppers (e.g. CAT)	|__|__|
j) Prescription drugs <u>not prescribed to you,</u> to get high (e.g., headache pills, codeine, trampolines).	|__|__|
k) Other drugs (e.g. glue, benzine) SPECIFY_______________	|__|__|

IF ALL D7=0 SKIP TO D9

D8. **(Modified – Couples)** Which days of the week do you typically use drugs (including dagga)?

(CHECK ALL THAT APPLY)
Monday 1
Tuesday 2
Wednesday 3
Thursday 4
Friday 5
Saturday... 6
Sunday... 7

D8a. **(Modified - RBA)** When was the last time you used drugs (including dagga)?
DAY.. |__|__|
MONTH.. |__|__|
YEAR ....|__|__|__|__|

D8b. What drugs did you use this last time? CHECK ALL THAT APPLY

Marijuana (dagga/cannabis/paji/zolo/weed/ganja/ntsango)........................ ❑1
Tik (Methamphetamine).. ❑2
Cocaine (coke, stone, rock) that you injected or snorted .... ❑3
Heroin.. ❑4
Nyaope/Thinka .... ❑5
Mandrax (buttons, white pipe, pille) .... ❑6
Inhalants (glue, petrol).. ❑7
Ecstasy (E), Club Drugs .... ❑8
Other amphetamines or uppers (e.g. CAT)... ❑9
Prescription drugs <u>not prescribed to you,</u> to get high
(e.g., headache pills, codeine, trampolines) .... ❑10
Other drugs (e.g. glue, benzine), SPECIFY___________ .... ❑99

D9. (Modified – RBA) Have you <u>ever</u> injected any drugs (with a needle)?
YES .... ❑1
NO .... ❑2 **(GO TO D16)**

D10. (RBA) During the past 3 months, about how many times did you inject drugs?
|__|__|__| .... TIMES [0-999]

D11. What was/were the last drug(s) you injected? CHECK ALL THAT APPLY
Heroin .... ❑1
Cocaine .... ❑2
Whoonga .... ❑3
Nyaope .... ❑4
Other .... ❑99
SPECIFY________________

D12. (Modified -RBA) Do you share needles?
YES .... ❑1
NO .... ❑2

D13. (Modified – RBA) Do you clean needles before you use them?
YES .... ❑1
NO .... ❑2

D14. (New?) Have you ever used a needle exchange?
YES .... ❑1
NO .... ❑2

D15. (New) Have you ever done Flash Blood or Bluetooth (sharing blood from a needle with another person using drugs)?
YES .... ❑1
NO .... ❑2

D16. (Modified – RBA) Do you know if your main male partner (*boyfriend, bae, husband*) has ever injected drugs?

No main male partner .... ❑0
YES .... ❑1
NO .... ❑2

D17. (Modified - WHC+) Has a health provider ever told you that you have Hepatitis C (Hep C – a liver disease)?

YES .... ❑1
NO .... ❑2

SECTION E: NEED FOR ALCOHOL AND/OR DRUG TREATMENT

We would like to know about your need for rehab and what you may know about it. Treatment or rehab is a place where you can go to get help to stop using drugs (including dagga) and alcohol.

E1. (PRBA – Baseline Part 1 - Modified) Do you think you have an alcohol or drug problem?

YES .... ❑1
NO .... ❑2

E2. (PRBA – Baseline Part 1 – Modified) Do you know where to go to get alcohol or drug treatment/rehab if you need it?

YES .... ❑1
NO .... ❑2

E3. (PRBA – Baseline Part 1) Do you want to be referred to alcohol or drug treatment/rehab now?

YES .... ❑1
NO .... ❑2

NOTE: Referral offered if E3 = YES.

SECTION F: DEPRESSION & ANXIETY

Now we would like to know about your emotions and mental health. Please respond with how you have felt in the past 3 months.

F1. **(Modified – TCU ADOL PSYFORM)** In the past 3 months, how often have you:

		Never	Rarely	Some-Times	Often	Almost Always
a.	Felt sad or depressed	1	2	3	4	5
b.	Had thoughts of committing suicide (killing yourself)	1	2	3	4	5
c.	Felt lonely	1	2	3	4	5
d.	Felt interested in life	1	2	3	4	5
e.	Felt hopeful about the future	1	2	3	4	5
f.	Felt extra tired or run down	1	2	3	4	5
g.	Worried a lot	1	2	3	4	5

F2. **(Modified – TCU ADOL PSYFORM)** In the past 3 months, how often have you:

		Never	Rarely	Some-Times	Often	Almost Always
a.	Had trouble sitting still for long	1	2	3	4	5
b.	Had trouble sleeping (woke-up a lot)	1	2	3	4	5
c.	Felt anxious or nervous	1	2	3	4	5
d.	Had trouble concentrating or remembering things	1	2	3	4	5
e.	Felt afraid of certain things, like lifts, crowds, or going out alone	1	2	3	4	5
f.	Felt tense or wound-up	1	2	3	4	5
g.	Felt tightness or tension in your muscles	1	2	3	4	5

NOTE: Referral offered if responses of 4 or 5 on F1a and/or F1b.

SECTION G: POST TRAUMATIC STRESS

Sometimes things happen to people that are especially frightening, horrible, or painful. For example:

- ***a physical or sexual assault or abuse***
- ***having an experience where you almost died***
- ***seeing someone be killed or seriously injured***
- ***having a loved one die***

G1. **(Modified – PC-PTSD-5)** Have you ever experienced anything particularly frightening, horrible, or traumatic?

YES ....❑1
NO ....❑2 **(GO TO SECTION H)**

IF G1=DK SKIP TO SEC H
IF G1=RF SKIP TO G3

Now we want to know about how you feel about this event. We understand that you may find it difficult to answer these questions, but please answer as much as you can.

G2a. (Modified Time – PC-PTSD-5) In the past 3 months have you had nightmares about the event(s) or thought about the event(s) when you did not want to?

YES ....❑1
NO ....❑2

G2b. (Modified Time– PC-PTSD-5) In the past 3 months have you tried hard not to think about the event(s) or went out of your way to avoid situations that reminded you of the event(s)?

YES ....❑1
NO ....❑2

G2c. (Modified Time – PC-PTSD-5) In the past 3 months have you been constantly on guard, watchful, or easily startled?

YES ....❑1
NO ....❑2

G2d. (Modified – PC-PTSD-5) In the past 3 months have you felt numb or detached from (not connected to) people, activities, or your surroundings?

YES ....❑1
NO ....❑2

G2e. (Modified Time - PC-PTSD-5) In the past 3 months have you felt guilty or unable to stop blaming yourself or others for the event(s) or any problems the event(s) may have caused?

YES ....❑1
NO ....❑2

G3. (NEW) Thank you for answering these questions. We understand they are sensitive and may have upset you. Would you like a referral for services?

YES ....❑1
NO...❑2

NOTE: Referral offered if three or more of G2a to G2e = YES OR IF G3=YES

G_END. Thank you for answering these questions, we know they are asking for a lot of information. Please take a break now before continuing. (require password to continue)

SECTION H: SEXUAL HISTORY

Now, we would like you to share with us some of your sexual history and practices with males, including recent vaginal sex or anal sex (called bum sex). Remember that this information is kept private.

H1. **(Modified – NC Clinics)** The first time you had sex with a male, what kind of sex was it?
Vaginal……………………………………………………………....❑1
Anal (bum)………………………………………………………….❑2

H2a. (Modified – PRBA) The first time you had sex with a male, was a condom used?
YES .... ❑1
NO .... ❑2

H2b. **(Modified – NC Clinics)** The first time you had sex with a male, were you high on alcohol or drugs (including dagga)?
YES .... ❑1
NO .... ❑2

H2c. (Modified – PRBA) How old were you when you had sex with a male for the first time?
AGE ....|__|__| [CHECK: H2c ≤ A1] [0-24]

H2d. (Modified – PRBA) Which of the following statements most closely describes your experience the first time you had sex with a male?
I was willing and consented 1
I was persuaded 2
I was tricked 3
I was forced/raped 4

Now, we hope you will answer the next questions about the times that you had sex with males because you wanted to, not pressured or forced. We would like you to share experiences with <u>any type</u> of male partner. Remember that this information is kept private.

First, we are going to talk about when you had vaginal or anal sex with any male, because you wanted to, in the past 3 months (90 days).

H4ax. **(Modified - Metro)** How many males did you have sex with in the last 3 months? What is your best guess?
OF PEOPLE..................|__|__|__| [0-999]

**ELIGIBILITY CHECK: IF H4ax=0 THEN ASK H4aCn; ELSE SKIP TO H4bx**

H4aCn. Just to confirm, how many males did you have sex with in the last 3 months?
.... |__|__|__| [0-999]

If H4aCn is NOT zero then goto H4b; else ask InelH4a:

InelH4a. Please alert a staff member for assistance. ___________
Staff must enter password (“prepare”, not case sensitive) to end interview as ineligible.

H4bx. **(Modified -Metro)** How many times did you have vaginal sex in the last 3 months? What is your best guess?

OF TIMES |__|__|__| [0-999]

IF H4bx=0 SKIP TO H4dx

H4cx. **(Modified -Metro)** How many times did you or your partner use a condom when you had vaginal sex in the last 3 months? What is your best guess?

OF TIMES |__|__|__| [CHECK if H4cx≤H4bx] [0-999]

H4dx. **(Modified -Metro)** How many times did you have anal sex in the last 3 months? What is your best guess?

OF TIMES |__|__|__| [0-999]

IF H4dx=0 SKIP TO H5

H4ex. **(Modified -Metro)** How many times did you or your partner use a condom when you had anal sex in the last 3 months? What is your best guess?

OF TIMES |__|__|__| [CHECK if H4ex≤H4dx] [0-999]

Now we want to know about sexual relationships with a main male partner (boyfriend, bae, husband). This is your 'main man,' 'straight partner' or ngqo. We hope you will answer about times that you had sex because you wanted to, not pressured or forced.

H5. In the past 3 months, have you had a main male partner (boyfriend, husband)?
YES 1
NO...2 **(GO TO H18x)**

H6. In the past 3 months, have you had sex with a main male partner (boyfriend, husband)?
YES 1
NO...2 **(GO TO H18x)**

IF H6=DK or RF SKIP TO H18x

H8. How old is your main male partner? If you are not sure, what is your best guess?
AGE.................................. |__|__| [10-80]

H9. How long have you been together with your main male partner?
HOW MANY YEARS ....|__|__| [0-24] [CHECK: YEARS ≤ A1
HOW MANY MONTHS......................|__|__| [0-11] and YEARS ≤ H8]
HOW MANY DAYS...........................|__|__| [0-30]

H10. **(Modified – NC Clinics)** How likely do you think it is that your main male partner is having sex with someone else? Would you say he definitely is, probably is, probably is not, or definitely is not?
Definitely is ....❑1
Probably is...❑2
Probably is not..❑3
Definitely is not ....❑4

H11. Have you talked with your main male partner about his HIV status?
YES ....❑1
NO...❑2

H11a. Have you talked with your main male partner about **<u>your</u>** HIV status?

YES .... ❑1
NO .... ❑2

H11b. Do you know your main male partner's HIV status?

Yes, he is negative .... ❑9 **(GO TO H12a)**
Yes, he is positive .... ❑1
No, I don't know .... ❑2 **(GO TO H12a)**

H11c. To the best of your knowledge, is your main male partner taking ARVs?

YES .... ❑1
NO .... ❑2
I DON'T KNOW .... ❑-7

H12a. The last time you had sex with your main male partner did you use a condom?

YES .... ❑1
NO .... ❑2

H13x. During the past 3 months, how many <u>times</u> did you have vaginal sex with your main male partner? What is your best guess?
OF TIMES .... |__|__|__| **(IF 0, GO TO H15x)** [0-999]

H14x. Of these times you had vaginal sex with your main male partner, how many times was a condom used? What is your best guess?
OF TIMES .... |__|__|__| [CHECK if H14x≤H13x] [0-999]

H15x. During the past 3 months, how many <u>times</u> did you have anal (bum) sex with your main male partner? What is your best guess?
OF TIMES .... |__|__|__| **(IF 0, GO TO H17)** [0-999]

H16x. Of these times you had anal (bum) sex with your main male partner, how many times was a condom used? What is your best guess?
OF TIMES .... |__|__|__| [CHECK if H16x≤H15x] [0-999]

H17. In the past 3 months, did you use alcohol, dagga, or other drugs just before or during sex with your main male partner?

YES .... ❑1
NO .... ❑2

Next, we would like to know about sex with other male sex partners. We are again talking about vaginal and anal (bum) sex because you wanted to have sex. These people are male sex partners who are <u>not</u> your main partner (boyfriend, husband). These partners can be friends, strangers, trading partners, casual partners, and some people call these blessors, under cover lovers, roll-ons, one night stands, 'ministers of finance', or 'ministers of transport'. Remember, these are NOT your main male partner (boyfriend or husband).

H18x. How many other partners did you have in the last 3 months? What is your best guess?
OF OTHER PARTNERS.......... |__|__|__| **(IF 0, GO TO Eligibility Check)** [0-999]

H19x. During the past 3 months, how many <u>times</u> did you have vaginal sex with other partners? What is your best guess?
OF TIMES ....|__|__|__| **(IF 0, GO TO H21x)** [0-999]

H20x. Of these times you had vaginal sex with other partners, how many times was a condom used? What is your best guess?
OF TIMES.................... |__|__|__| [CHECK if H20x≤H19x] [0-999]

H21x. During the past 3 months, how many <u>times</u> did you have anal (bum) sex with other partners? What is your best guess?
OF TIMES ....|__|__|__| **(IF 0, GO TO Eligibility Check)** [0-999]

H22x. Of these times you had anal sex with other partners, how many times was a condom used? What is your best guess?
OF TIMES.................... |__|__|__| [CHECK if H22x≤H21x] [0-999]

**NOTE: ELIGIBILITY CHECKS**<br>**1) IF H13x+H15x+H19x+H21x=0**<br>**2) IF H13x=H14x AND H15x=H16x AND H19x=H20x AND H21x=H22x**

IF ANY OF H13x, H14x, H15x, H16x, H19x, H20x, H21x, OR H22x = DK OR RF SKIP TO H23

IF ((H13 + H15 + H19 + H21) = 0) OR
(H13=H14 & H15 =H16 & H19=H20 & H21=H22) ASK ScA4Cnx; ELSE SKIP TO H23

ScA4Cnx. Thank you for your responses, we know we are asking for a lot of information. Please confirm your answer. Have you had sex, either vaginal or anal sex, without a condom with a male partner in the last 3 months?
YES .... ❑1
NO.. ❑2
[DO NOT ALLOW DK OR RF RESPONSES]

IF ScA4Cn = NO ASK InelH22; ELSE SKIP TO H23

InelH22x. Please alert a staff member for assistance. ___________
Staff must enter password (“prepare”, not case sensitive) to end interview as ineligible.

IF H18=0 GO TO SECTION I

H23. The last time you had sex with one of your other partners, did you use a condom?
YES .... ❑1
NO.. ❑2

H24. In the past 3 months, did you use alcohol, dagga, or other drugs just before or during sex with one of your other partners?
YES .... ❑1
NO.. ❑2

SECTION I: TRANSACTIONAL SEX

Sometimes women find themselves in situations where they trade sex to get things they want or need. Please answer the next questions about times when you exchanged sex for things you needed or wanted. This can include oral sex (blow jobs) as well as vaginal or anal sex. Remember that this information is kept private.

I1. Have you ever traded sex to get the things you need (like drugs, money, food, clothing, shelter, transport, good grades, or any other things) with your main male sexual partner, casual partners, or formal sex trading partners (clients)?

YES .... ❑1
NO .... ❑2

IF I1 = NO, DK, RF (GO TO SECTION J)

**IF H5 or H6 = NO, DK, or RF SKIP TO I1b**

I1a. Have you traded sex to get the things you need with your main male sexual partner in the past 3 months?

YES .... ❑1
NO .... ❑2

I1b. Have you traded sex to get the things you need with other partners (not clients or your main male partner) in the past 3 months?

YES .... ❑1
NO .... ❑2

I1c. Have you traded sex to get the things you need with formal sex trading partners (clients) in the past 3 months?

YES .... ❑1
NO .... ❑2

**IF H5 or H6 = NO, DK, or RF SKIP TO I3**<br>**IF I1a = NO, DK, or RF SKIP TO I3**

I2. Please check all that apply, in the past 3 months, have you had sex with your main male partner to get...?

	**YES**	**NO (skip to next item)**
a. Food	❑1	❑2
a1. How many times in the past 3 months did you have sex with your main male partner to get food? |__|__|__| [1-999]		
b. Drugs or alcohol	❑1	❑2
b1. How many times in the past 3 months…? |__|__|__| [1-999]		
c. Clothing	❑1	❑2
c1. How many times in the past 3 months…? |__|__|__| [1-999]		
d. Money	❑1	❑2
d1. How many times in the past 3 months…? |__|__|__| [1-999]		
e. A place to stay	❑1	❑2
e1. How many times in the past 3 months…? |__|__|__| [1-999]		
f. Items or money for your family or children	❑1	❑2

f1. How many times in the past 3 months…?. |__|__|__| [1-999]

g. School fees .... ❑1 .... ❑2

g1. How many times in the past 3 months…? |__|__|__| [1-999]

h. Cell phone (air time) .... ❑1 .... ❑2

h1. How many times in the past 3 months…? |__|__|__| [1-999]

i. Good grades .... ❑1 .... ❑2

i1. How many times in the past 3 months…?. |__|__|__| [1-999]

j. Beauty products (e.g., hair supplies, makeup) .... ❑1 .... ❑2

j1. How many times in the past 3 months…?. |__|__|__| [1-999]

k. Anything else .... ❑1 .... ❑2

Specify the "other" things you traded sex with your main male partner to get________________________

k1. How many times in the past 3 months…? .... |__|__|__| [1-999]

I2l. In the past 3 months, was oral sex (blow job) something you gave to your main male partner to get things you needed?

YES .... ❑1

NO .... ❑2

IF I1b = NO, DK, or RF SKIP TO I4

I3. Please check all that apply, in the past 3 months, have you had sex with your other partners (not clients or your main male partner) to get...? **YES** **NO (skip to next item)**

a. Food .... ❑1 .... ❑2

a1. How many times in the past 3 months did you have sex with other partners to get…? .... |__|__|__| [1-999]

b. Drugs or alcohol .... ❑1 .... ❑2

b1. How many times in the past 3 months…? |__|__|__| [1-999]

c. Clothing .... ❑1 .... ❑2

c1. How many times in the past 3 months…? |__|__|__| [1-999]

d. Money .... ❑1 .... ❑2

d1. How many times in the past 3 months…? |__|__|__| [1-999]

e. A place to stay .... ❑1 .... ❑2

e1. How many times in the past 3 months…? |__|__|__| [1-999]

f. Items or money for your family or children .... ❑1 .... ❑2

f1. How many times in the past 3 months…?. |__|__|__| [1-999]

g. School fees .... ❑1 .... ❑2

g1. How many times in the past 3 months…? |__|__|__| [1-999]

h. Cell phone (air time) .... ❑1 .... ❑2

h1. How many times in the past 3 months…? |__|__|__| [1-999]

i. Good grades .... ❑1 .... ❑2

i1. How many times in the past 3 months…?. |__|__|__| [1-999]

j. Beauty products (e.g., hair supplies, makeup) .... ❑1 .... ❑2

j1. How many times in the past 3 months…?. |__|__|__| [1-999]

k. Anything else .... ❑1 .... ❑2

Specify the "other" things you traded sex with other partners to get________________________

k1. How many times in the past 3 months…? |__|__|__| [1-999]

I3l. In the past 3 months, was oral sex (blow job) something you gave to other partners to get things you needed?

YES .... ❑1
NO .... ❑2

**IF I1c = NO, DK, or RF SKIP TO SECTION J**

I4. Please check all that apply, in the past 3 months, have you had sex with formal sex trading partners (sex clients) to get...?

	**YES**	**NO (skip to next item)**
a. Food	❑1	❑2
a1. How many times in the past 3 months did you have sex with formal sex trading partners to get…? .... |__|__|__| [1-999]		
b. Drugs or alcohol	❑1	❑2
b1. How many times in the past 3 months…? |__|__|__| [1-999]		
c. Clothing	❑1	❑2
c1. How many times in the past 3 months…? |__|__|__| [1-999]		
d. Money	❑1	❑2
d1. How many times in the past 3 months…? |__|__|__| [1-999]		
e. A place to stay	❑1	❑2
e1. How many times in the past 3 months…? |__|__|__| [1-999]		
f. Items or money for your family or children	❑1	❑2
f1. How many times in the past 3 months…?. |__|__|__| [1-999]		
g. School fees	❑1	❑2
g1. How many times in the past 3 months…? |__|__|__| [1-999]		
h. Cell phone (air time)	❑1	❑2
h1. How many times in the past 3 months…? |__|__|__| [1-999]		
i. Good grades	❑1	❑2
i1. How many times in the past 3 months…?. |__|__|__| [1-999]		
j. Beauty products (e.g., hair supplies, makeup)	❑1	❑2
j1. How many times in the past 3 months…?. |__|__|__| [1-999]		
k. Anything else	❑1	❑2
Specify the "other" things you traded sex with clients to get_________________		
k1. How many times in the past 3 months…?....... |__|__|__| [1-999]		

I4l. In the past 3 months, was oral sex (blow job) something you gave to clients to get things you needed?

YES .... ❑1
NO .... ❑2

SECTION J: CONDOM NEGOTIATION & SEXUAL DISCUSSION

IF H5=No, DK, or RF, or H6 (sex with a main male partner) = NO, DK, or RF SKIP TO J2

Now we want to know about sexual communication with your main male partner, with whom you regularly have sex. We would like to know about your most recent main male partner.

**J1.** In the past 3 months how often did you...	**Not at All**	**Some-times**	**Many Times**	**All the Time**
a) Ask your main male partner to use a male condom?	❑1	❑2	❑3	❑4
b) Ask your main male partner about using a female condom?	❑1	❑2	❑3	❑4
c) Use a condom even if you were high or drunk?	❑1	❑2	❑3	❑4
d) Use a condom even if your main male partner was high or drunk?	❑1	❑2	❑3	❑4
e) Ask how many other sex partners your main male partner has had?	❑1	❑2	❑3	❑4
f) Ask your main male partner if he wants any children	❑1	❑2	❑3	❑4
g) Discuss pregnancy prevention with your main male partner	❑1	❑2	❑3	❑4

Now we want to know about condom communication with <u>all partners.</u>

J2. Did you have trouble proposing condom use in the past 3 months?

YES .... ❑1
NO, I didn't propose condoms.. ❑2 **(GO TO SECTION K)**
NO, I did not have trouble proposing condoms with my sexual partners ❑3 **(GO TO SECTION K)**

J3. With which partners did you have trouble proposing condom use in the past 3 months?
(CHECK ALL THAT APPLY)

Main male partner 1
Other sexual partner.. 2
Sex trading partner (sex client)... 3

SECTION K: POWER AND EMPOWERMENT

IF H5 = NO, DK, or RF, or H6 = NO, DK, or RF SKIP TO SECTION L

The following questions concern your sexual relationship with your main male partner. This is 'your man' or a man that you are in a relationship with and could also be a friend, boyfriend, husband, or a spouse.

**K1.** Please answer whether you strongly disagree, disagree, agree, or strongly agree with the following statements:	**Strongly Disgree**	**Disgree**	**Agree**	**Strongly Agree**
a) Sometimes you have sex with your main male partner even when you do not want to because it is expected of you.	❑1	❑2	❑3	❑4
b) Sometimes you have sex with your main male partner even when you do not want to because you are afraid to say NO.	❑1	❑2	❑3	❑4
c) If you refuse to have sex with your main male partner, he will refuse to give you money or pay the bills.	❑1	❑2	❑3	❑4
d) If you refuse to have sex with your main male partner, he will stop giving you his love.	❑1	❑2	❑3	❑4
e) If you refuse to have sex with your main male partner, he will become angry or shout at you.	❑1	❑2	❑3	❑4

SECTION L: ENVIRONMENT

We would like you to share some information about your community. Please tell us about your experiences during the past 3 months (90 days).

L1a. ***(AM – Salinas)*** How safe from violence have you felt in your community during the past **3 months**?

Very safe ❑1
Safe ❑2
Unsafe ❑3
Very unsafe ❑4

L1b. ***(AM – Salinas)*** In the past **3 months**, how often did you limit the places you went to in your community because you were concerned for your safety?

Never ❑1
Once ❑2
Sometimes ❑3
Often ❑4

**L2**. During the past **3 months** in the community surrounding your house, how often was there…?	**NEVER** ▼	**RARELY** ▼	**SOME-TIMES** ▼	**OFTEN** ▼
a. Drinking in public?	1	2	3	4
b. People selling or using drugs?	1	2	3	4
c. A car stolen?	1	2	3	4
d. A burglary (house break-in)?	1	2	3	4
e. A violent argument between neighbors?	1	2	3	4
f. An arrest in public?	1	2	3	4
g. Arson (the intentional setting of a house or building on fire)?	1	2	3	4
h. Audible gun shots?	1	2	3	4
i. A fight in which a weapon like a gun or knife was used?	1	2	3	4
j. A gang fight?	1	2	3	4
k. A sexual assault or rape?	1	2	3	4
l. A robbery or mugging?	1	2	3	4
m. . A drive-by shooting?	1	2	3	4
n. A murder?	1	2	3	4

L3. Which of the following do you have in your community?
(CHECK ALL THAT APPLY)

Library .... ❑1
Community Centre.. ❑2
Secondary School .... ❑3
Healthcare Clinics .... ❑4
Public, 24 Hour, Healthcare Clinics .... ❑5
Church... ❑6
Public Park or Playground .... ❑7
Technical Training School .... ❑8
Youth Friendly Clinic.. ❑9
WiFi Hotspots .... ❑10
Grocery Stores .... ❑11
Tuc Shops .... ❑12
Shopping Centre .... ❑13
Multipurpose Centre .... ❑14
Other .... ❑99
Specify other locations in your community________________________
None .... ❑100

SECTION M: HIV TESTING AND RISK

Now we would like to know about HIV testing. Again, your answers will be kept confidential and private.

M1. Have you ever been tested for HIV (not including the testing that you had when you joined the study)?

YES .... ❑1
NO .... ❑2 **(GO TO M3)**

M2. How many times have you been tested for HIV altogether?

OF TIMES .... |__|__| [1-99]

Now we would like to know about your perceptions of HIV risk. Again, your answers will be kept confidential and private.

M3. How would you describe your chances of getting HIV in the next year?

No risk at all ❑1 **(GO TO Section N)**
Small chance ❑2
Moderate chance ❑3
Great chance ❑4

M4. Why do you think you are at risk?
(CHECK ALL THAT APPLY)

You do not use condoms all the time .... ❑1
Your main male partner does not use condoms all the time .... ❑2
You inject drugs with other people .... ❑3
Your main male partner injects drugs with other people .... ❑4
Sexual assault (rape) .... ❑5
Other.. ❑99
Specify other reasons you think you are at risk for HIV
____

SECTION N: WOMEN'S HEALTH

In this section, we would like to know about your well-being (including general health, symptoms you may experience, menstrual [your period] health, and reproductive health).

N1. Did anyone tell you about menstruation (your period) before you started menstruating?
YES ....❑1
NO..❑2 **(GO TO N3)**

N2. Who educated you regarding menstruation (your period)?
(CHECK ALL THAT APPLY)
Mother ....❑1
Teacher ....❑2
Friends ....❑3
Books ....❑4
Media (TV, Radio, Internet) ....❑5
Nurse ....❑6
NGO..❑7
Others (Specify)_______________ ....❑99

N3. Have you ever been taught to care for (clean) your vagina?
YES ....❑1
NO..❑2

N3a. What do you use to clean yourself?
(CHECK ALL THAT APPLY)
Water and soap ....❑1
Water only ....❑2
Tissue paper ....❑3
Towel ....❑4
Others (Specify)_______________ ....❑99

Now, we would like you to share some information with us about preventing pregnancy, or your use of birth control. That is, we are asking about what you are using to keep from falling pregnant.

N4. Have you ever discussed methods to avoid falling pregnant with your sexual partners (main male or other)?
YES ....❑1
NO ....❑2 **(GO TO N5)**

N4a. In the past 3 months have you discussed methods to avoid pregnancy with your sexual partners (main male or other)?
YES ....❑1
NO ❑2

N5. Are you on birth control now (a method to keep from falling pregnant)?
YES ....❑1 **(GO TO N6)**
NO ....❑2

IF N5=DK or RF SKIP TO N7

N5a. Why are you not using birth control now?
(CHECK ALL THAT APPLY)
Stigma from the clinics (judging and shaming from staff) ❑1
Shame from family members ❑2
Main male partner did not want me to use birth control ❑3
Cost ❑4
Transportation ❑5
Understanding and filling out the medical forms ❑6
Afraid I will see someone I know ❑7
Don't know where to get pregnancy prevention method ❑8
Side effects ❑9
Local clinics don't have needed services ❑10
I want to get pregnant ❑11
I did not have time to go to clinics ❑12
Lack of childcare ❑13
My main male partner wants a child ❑14
Something else? ❑99
Specify other reasons you didn't use birth control ____________________

IF N5 = NO, SKIP TO N7

N6. What are you doing now to prevent pregnancy (keep from falling pregnant)?
(CHECK ALL THAT APPLY)
Oral contraceptives ("the pill") ❑1
Implant ❑2
Injection (Depo, Nur-Isterate) (the shot) ❑3
IUD/Loop ❑4
Male condoms ❑5
Female condoms ❑6
Withdrawal ❑7
Rhythm method ❑8
Traditional medicine (muti) ❑9
Something else? ❑99
Specify____

Now, we would like to know about your current or past pregnancies and antenatal health.

N7. Have you ever been pregnant? This includes miscarriages, abortions, stillbirth (a baby born dead), and giving birth.
YES ❑1
NO ❑2 **(GO TO N14)**

N8. How old were you the first time you became pregnant?
AGE |__|__| [5-24]

N9. How many times have you been pregnant?
OF TIMES |__|__| [1-10]

N10. How many children have you given birth to?
OF CHILDREN |__|__| [0-10] **IF 0 GO TO N12**

N11. Have you received any SASSA social grant (money) for having children?

YES.. 1
NO... 2

N12. Have you ever been to antenatal care (that is, care for pregnancy)?
YES .... ❑1
NO... ❑2

N12a. Have you ever stayed away from antenatal care because you were afraid that clinic staff would judge or shame you?

YES.. 1
NO... 2

N12b. The last time you were pregnant, did you avoid antenatal care because you were judged or shamed by clinic staff in the past?

YES.. 1
NO... 2

N13. **(WHO MCS)** Was there ever a time when you were beaten or physically assaulted by (any of) your sexual partner(s) while you were visibly pregnant?
YES .... ❑1
NO... ❑2

Finally, we would like to know about your physical health.

**N14.** In the past 3 months, have you:		**YES**	**NO**
a)	Experienced any burning or pain when passing urine	❑1	❑2
b)	Had any unusual smelly discharge or drip from your vagina	❑1	❑2
c)	Had any painful sores on your vagina	❑1	❑2
d)	Had sores on your vagina that didn't hurt	❑1	❑2
e)	Had any warts on your vagina (cauliflower)	❑1	❑2
f)	Had dark, foamy urine	❑1	❑2

NOTE: IF PARTICIPANT RESPONDS "YES," TO ANY SYMPTOM IN N14, REFER TO A HEALTH CLINIC.

N15. In general, would you say your health is:
Excellent.. ❑1
Very good... ❑2
Good .... ❑3
Fair.. ❑4
Poor .... ❑5

SECTION O: LAST SEXUAL EPISODE

Now, please think about the LAST TIME that you had vaginal or anal sex with a male partner.

O1. The very last time you had sex, what kind was it?
Vaginal .... ❑1
Anal (bum)... ❑2

O2. When was the last time you had sex?
Within the past two days .... ❑1
3-6 days ago .... ❑2
1-3 weeks ago .... ❑3
1-3 months ago... ❑4
4-12 months ago... ❑5
More than 12 months ago .... ❑6
Never.. ❑7

NOTE: ELIGIBILITY CHECK IF O2 = 5, 6 OR 7

IF O2 = 5, 6, 7, DK or RF, ASK O2Cn; ELSE SKIP TO O3

O2Cn. Just to confirm, when was the last time you had sex?
Within the past two days .... ❑1
3-6 days ago... ❑2
1-3 weeks ago .... ❑3
1-3 months ago... ❑4
4-12 months ago... ❑5
More than 12 months ago .... ❑6
Never.. ❑7
[DO NOT ALLOW DK OR RF RESPONSES]

IF O2Cn = 5, 6 or 7 ASK IneIO2; ELSE SKIP TO O3

IneIO2. Please alert a staff member for assistance. ___________
Staff must enter password (“prepare”, not case sensitive) to end interview as ineligible.

O3. Who was the last sex act with?
Main male partner 1
Other sexual partner...2
Sex trading partner (sex client).....................................3

O4. This last time you had sex, did you use a condom?
YES .... ❑1
NO.. ❑2

O5. This last time you had sex, did you use drugs (including dagga) or alcohol just before or during sex?

YES .... ❑1
NO .... ❑2

O6. Did this partner use drugs (including dagga) or alcohol just before or during sex that last time?

YES .... ❑1
NO .... ❑2

O7. Which of the following statements most closely describes your experiences when you last had sex?

I was willing and consented 1
I was persuaded 2
I was tricked 3
I was forced/raped 4

**NOTE: REFERRAL O7 = 3 OR 4**

SECTION P: VICTIMISATION

IF H5 = NO, DK, or RF, or H6 = NO, DK, or RF, SKIP TO P2

We want you to share a little more about your relationship with your main male partner, how you think he would react in certain situations, and things he might have done to you recently (in the past 3 months). Remember this information will remain strictly confidential.

P1. The following questions concern your sexual relationship with your **main male partner**. Please respond whether you ***strongly agree, agree, neither agree nor disagree (neutral), disagree, or strongly disagree*** with the following statements:

	Strongly Agree ▼	Agree ▼	Neither agree nor disagree (neutral) ▼	Disagree ▼	Strongly Disagree ▼
a. If you refuse to have sex with your main male partner he will beat you.	1	2	3	4	5
b. If you refuse to have sex with your main male partner he will rape you	1	2	3	4	5
c. If you refuse to have sex with your main male partner he will send friends to rape you	1	2	3	4	5

(WHO MULTI-COUNTRY STUDY – WITH MODIFICATIONS) ***The next questions are about things that happen to many women, and that your current main male sexual partner (husband or boyfriend), or any other sexual partner may have done to you.***

**P2.** **(WHO MCS - MOD)** I want you to tell me if your current main male partner, or any other partner, has ever done the following things to you. Please check all that apply, has any sexual partner ever…	**YES**	**NO**
a) **(WHO MCS)** Insulted you or made you feel bad about yourself?	**Part of CHECK ALL THAT APPLY**	
a1) **(NEW)** Which partner insulted you or made you feel bad about yourself? Check all that apply. 1=Main male partner, 2=Other partner		
a2) **(WHO MCS – MOD)** Has any partner insulted you or made you feel bad about yourself in the past 3 months?	❑1	❑2 SKIP P2a3
a3) **(NEW)** Which partner insulted you or made you feel bad about yourself in the past 3 months? Check all that apply. 1=Main male partner, 2=Other partner		
b) **(WHO MCS - MOD)** Belittled or humiliated you (made you feel small) in front of other people?	**Part of CHECK ALL THAT APPLY**	
b1) **(NEW)** Which partner belittled or humiliated you (made you feel small) in front of other people? Check all that apply. 1=Main male partner, 2=Other partner		
b2) **(WHO MCS – MOD)** Has any partner belittled or humiliated you (made you feel small) in front of other people in the past 3 months?	❑1	❑2 SKIP P2b3
b3) **(NEW)** Which partner belittled or humiliated you (made you feel small) in front of other people in the past 3 months? Check all that apply. 1=Main male partner, 2=Other partner		

c) **(WHO MCS)** Done things to scare or intimidate you on purpose (e.g., by the way he looked at you, by yelling and smashing things)?	**Part of CHECK ALL THAT APPLY**
c1) **(NEW)** Which partner did things to scare or intimidate you on purpose (e.g., by the way he looked at you, by yelling and smashing things)? Check all that apply. 1=Main male partner, 2=Other partner	
c2) **(WHO MCS – MOD)** Has any partner done things to scare or intimidate you on purpose (e.g., by the way he looked at you, by yelling and smashing things) in the past 3 months?	❑1 ❑2 SKIP P2c3
c3) **(NEW)** Which partner did things to scare or intimidate you on purpose (e.g., by the way he looked at you, by yelling and smashing things) in the past 3 months? Check all that apply. 1=Main male partner, 2=Other partner	
d) **(WHO MCS)** Threatened to hurt you or someone you care about?	**Part of CHECK ALL THAT APPLY**
d1) **(NEW)** Which partner threatened to hurt you or someone you care about? Check all that apply. 1=Main male partner, 2=Other partner	
d2) **(WHO MCS – MOD)** Has any partner threatened to hurt you or someone you care about in the past 3 months?	❑1 ❑2 SKIP P2d3
d3) **(NEW)** Which partner threatened to hurt you or someone you care about in the past 3 months? Check all that apply. 1=Main male partner, 2=Other partner	

**P3*.*** **(WHO MCS)** Please check all that apply, has your main male partner or any other partner ever…	**YES NO**
a) **(WHO MCS)** Slapped you or thrown something at you that could hurt you?	**Part of CHECK ALL THAT APPLY**
a1) **(NEW)** Which partner slapped you or threw something at you that could hurt you? Check all that apply. 1=Main male partner, 2=Other partner	
a2) **(WHO MCS – MOD)** Has any partner slapped you or threw something at you that could hurt you in the past 3 months?	❑1 ❑2 SKIP P3a3
a3) (NEW) Which partner slapped you or threw something at you that could hurt you in the past 3 months? Check all that apply. 1=Main male partner, 2=Other partner	
b) **(WHO MCS)** Pushed or shoved you?	**Part of CHECK ALL THAT APPLY**
b1) **(NEW)** Which partner pushed or shoved you? Check all that apply. 1=Main male partner, 2=Other partner	
b2) **(WHO MCS – MOD)** Has any partner pushed or shoved you in the past 3 months?	❑1 ❑2 SKIP P3b3
b3) (NEW) Which partner pushed or shoved you in the past 3 months? Check all that apply. 1=Main male partner, 2=Other partner	
c) **(WHO MCS)** Hit you with his fist or with something else that could hurt you?	**Part of CHECK ALL THAT APPLY**
c1) **(NEW)** Which partner hit you with his fist or with something else that could hurt you? Check all that apply. 1=Main male partner, 2=Other partner	
c2) **(WHO MCS – MOD)** Has any partner hit you with his fist or with something else that could hurt you in the past 3 months?	❑1 ❑2 SKIP P3c3
c3) (NEW) Which partner hit you with his fist or with something else that could hurt you in the past 3 months? Check all that apply. 1=Main male partner, 2=Other partner	
d) **(WHO MCS)** Kicked you, dragged you or beaten you up?	**Part of CHECK ALL THAT APPLY**

d1) **(NEW)** Which partner kicked you, dragged you or beat you up? Check all that apply. 1=Main male partner, 2=Other partner		
d2) **(WHO MCS – MOD)** Has any partner kicked you, dragged you or beat you up in the past 3 months?	❑1	❑2 SKIP P3d3
d3) (NEW) Which partner kicked you, dragged you or beat you up in the past 3 months? Check all that apply. 1=Main male partner, 2=Other partner		
e) **(WHO MCS)** Choked you on purpose?	**Part of CHECK ALL THAT APPLY**	
e1) **(NEW)** Which partner choked you on purpose? Check all that apply. 1=Main male partner, 2=Other partner		
e2) **(WHO MCS – MOD)** Has any partner choked you on purpose in the past 3 months?	❑1	❑2 SKIP P3e3
e3) (NEW) Which partner choked you on purpose in the past 3 months? Check all that apply. 1=Main male partner, 2=Other partner		
f) **(WHO MCS)** Burnt you (with a cigarette or hot water) on purpose?	**Part of CHECK ALL THAT APPLY**	
f1) **(NEW)** Which partner burnt you (with a cigarette or hot water) on purpose? Check all that apply. 1=Main male partner, 2=Other partner		
f2) **(WHO MCS – MOD)** Has any partner burnt you (with a cigarette or hot water) on purpose in the past 3 months?	❑1	❑2 SKIP P3f3
f3) (NEW) Which partner burnt you (with a cigarette or hot water) on purpose in the past 3 months? Check all that apply. 1=Main male partner, 2=Other partner		
g) **(WHO MCS)** Threatened to use or actually used a gun, knife or other weapon against you?	**Part of CHECK ALL THAT APPLY**	
g1) **(NEW)** Which partner threatened to use or actually used a gun, knife or other weapon against you? Check all that apply. 1=Main male partner, 2=Other partner		
g2) **(WHO MCS – MOD)** Has any partner threatened to use or actually used a gun, knife or other weapon against you in the past 3 months?	❑1	❑2 SKIP P3g3
g3) (NEW) Which partner threatened to use or actually used a gun, knife or other weapon against you in the past 3 months? Check all that apply. 1=Main male partner, 2=Other partner		

**P4.** **(WHO MCS)** Please check all that apply, has your main male partner or any other partner ever…	**YES**	**NO**
a) **(WHO MCS)** …physically forced you to have sexual intercourse when you did not want to?	**Part of CHECK ALL THAT APPLY**	
a1) **(NEW)** Which partner physically forced you to have sexual intercourse when you did not want to? Check all that apply. 1=Main male partner, 2=Other partner		
a2) **(WHO MCS – MOD)** Has any partner physically forced you to have sexual intercourse when you did not want to in the past 3 months?	❑1	❑2 SKIP P4a3
a3) (NEW) Which partner physically forced you to have sexual intercourse when you did not want to in the past 3 months? Check all that apply. 1=Main male partner, 2=Other partner		
b) **(WHO MCS)** …made you have sexual intercourse you did not want because you were afraid of what he/they might do?	**Part of CHECK ALL THAT APPLY**	
b1) **(NEW)** Which partner made you have sexual intercourse you did not want because you were afraid of what he/they might do? Check all that apply. 1=Main male partner, 2=Other partner		

b2) **(WHO MCS – MOD)** Has any partner made you have sexual intercourse you did not want because you were afraid of what he/they might do in the past 3 months?	❑1	❑2 SKIP P4b3
b3) (NEW) Which partner made you have sexual intercourse you did not want because you were afraid of what he/they might do in the past 3 months? Check all that apply. 1=Main male partner, 2=Other partner		
c) **(WHO MCS)** …forced you to do something sexual that you found degrading or humiliating?	**Part of CHECK ALL THAT APPLY**	
c1) **(NEW)** Which partner forced you to do something sexual that you found degrading or humiliating? Check all that apply. 1=Main male partner, 2=Other partner		
c2) **(WHO MCS – MOD)** Has any partner forced you to do something sexual that you found degrading or humiliating in the past 3 months?	❑1	❑2 SKIP P4c3
c3) (NEW) Which partner forced you to do something sexual that you found degrading or humiliating in the past 3 months? Check all that apply. 1=Main male partner, 2=Other partner		

Now we would like to know about things that other people may have done to you. Remember this information will remain strictly confidential.

P5. When was the last time you were physically abused by anyone (including main male or other sex partners)?

Within the past two days....................1
3-6 days ago....................2
1-3 weeks ago....................3
1-3 months ago....................4
4-12 months ago....................5
More than 12 months ago....................6
Never....................7 **(GO TO P8a)**
REFUSED....................-9 **(GO TO P8a)**

P6. ***(Western Cape – MOD)*** The last time you were physically hurt, who hurt you?

Main male partner....................1
Other sexual partner....................2
Sex trading partner (client)....................3
Male relative....................4
Female relative....................5
Male Friend....................6
Female Friend....................7
Male Stranger....................8
Female Stranger....................9
Gang member....................10
Police....................11
Drug dealer....................12
Teacher....................13
Other....................99

**IF P5 = 5 OR 6 SKIP TO P8a**

P7. How many times in the past **3 months** have you been physically abused by anyone?
NUMBER OF TIMES.................... |__|__| [1-99]

P8a. When was the last time you were forced to have vaginal sex against your will?

Within the past two days.. 1
3-6 days ago.. 2
1-3 weeks ago.. 3
1-3 months ago.. 4
4-12 months ago.. 5
More than 12 months ago.. 6
Never ....7 **(GO TO P8c)**
REFUSED.. -9 **(GO TO P8c)**

P8b. ***(Western Cape – MOD)*** The last time you were pressured or forced to have vaginal sex against your will, who pressured or forced you?

Main male partner 1
Other sexual partner...2
Sex trading partner (client) 3
Male relative...4
Female relative...5
Male Friend 6
Female Friend 7
Male Stranger...8
Female Stranger...9
Gang member 10
Police 11
Drug dealer 12
Teacher..13
Other 99

P8c. When was the last time you were forced to have anal sex against your will?

Within the past two days.. 1
3-6 days ago.. 2
1-3 weeks ago.. 3
1-3 months ago.. 4
4-12 months ago.. 5
More than 12 months ago.. 6
Never ....7 **(GO TO P8e)**
REFUSED.. -9 **(GO TO P8e)**

P8d. ***(Western Cape – MOD)*** The last time you were pressured or forced to have anal sex against your will, who pressured or forced you?

Main male partner 1
Other sexual partner...2
Sex trading partner (client) 3
Male relative...4
Female relative...5
Male Friend 6
Female Friend 7
Male Stranger...8
Female Stranger...9
Gang member 10
Police 11
Drug dealer 12
Teacher..13
Other 99

P8e. When was the <u>last time</u> you were forced to have oral sex against your will?
Within the past two days.. 1
3-6 days ago... 2
1-3 weeks ago.. 3
1-3 months ago... 4
4-12 months ago.. 5
More than 12 months ago... 6
Never ....7 **(GO TO P9)**
REFUSED... -9 **(GO TO P9)**

P8f. ***(Western Cape – MOD)*** The <u>last time</u> you were pressured or forced to have oral sex against your will, who pressured or forced you?

Main male partner 1
Other sexual partner..2
Sex trading partner (client) 3
Male relative...4
Female relative..5
Male Friend 6
Female Friend 7
Male Stranger..8
Female Stranger...9
Gang member 10
Police 11
Drug dealer 12
Teacher...13
Other 99

If Never for all three of P8a, P8c, and P8e, skip to P10.

P9. How many times in your lifetime have you ever been forced to have any sex against your will?
NUMBER TIMES.. |__|__|

DK... -7
REFUSED.. -9

P10. How many times in the past **3 months** has anyone touched you sexually or forced you to engage in any sex acts against your will (including rape)?
NUMBER OF TIMES .... |__|__| [0-99]

DK ....-7
REFUSED ....-9

P11. Thank you for answering these questions. We understand they are sensitive and may have upset you. Would you like a referral for services?
YES .... ❑1
NO... ❑2

**NOTE:** Referral offered if P11 = YES, ELSE<br>Referral offered if P7 > 0 OR P9 > 0 OR P10 > 0 AND P11 DOES NOT EQUAL NO.

SECTION Q: SOCIAL SUPPORT & AGENCY

Now, we want to know about the people around you. Select the option that best describes how you think about these people in the following situations. If the statement doesn't apply to you please select N/A. For example, if you don't have a main male partner, or don't have friends you consider close, select N/A as your response.

Q3. In general, how supported do you feel by the adults in your life?

Almost never supported	❑1
Sometimes supported	❑2
Very well supported	❑3
N/A	❑99

Q4. In general, how supported do you feel by your close friends?

Almost never supported	❑1
Sometimes supported	❑2
Very well supported	❑3
N/A	❑99 **(GO TO Q8)**

Q5. I feel comfortable talking to my close friends about my sexual relationships.

Strongly disagree	❑1
Disagree	❑2
Agree	❑3
Strongly agree	❑4

Q6. I feel comfortable talking to my close friends about health-related issues.

Strongly disagree	❑1
Disagree	❑2
Agree	❑3
Strongly agree	❑4

Q7. In the past 3 months, did you ask a close friend for help (examples of help could include: relationship advice, health advice, to borrow money or other things)?

Yes	❑1
No	❑2 **(GO TO Q8)**

Q7a. In the past 3 months, when you asked a close friend for help, did they help you?

Yes	❑1
No	❑2

We are now going to ask you questions about people whom you might talk to about PrEP.

Q8. I feel comfortable talking to my close friends about my interest in PrEP.

Yes	❑1
No	❑2
N/A	❑99

Q9. I feel comfortable talking to my main male partner about my interest in PrEP.

Yes	❑1
No	❑2
N/A	❑99

Q10. I feel comfortable talking to my family about my interest in PrEP.

Yes ❑1
No ❑2
N/A ❑99

Q11. Would you be interested in taking PrEP when it becomes available?

Yes ❑1
No ❑2 **[GO TO SECTION R]**

Q12. Do you want to tell anyone about your interest in taking PrEP?

Yes ❑1
No ❑2 **[GO TO Q14]**

Q13.

Answer "yes" or "no" for each person(s) you plan to tell in the list below.	YES	NO	N/A
	▼	▼	▼
a. Your main male partner	1	2	99
b. Your other sex partners	1	2	99
c. Your mother	1	2	99
d. Your father	1	2	99
ex. Your sister or brother	1	2	99
i. Friends	1	2	99
jx. Other persons	1	2	99

Please specify who else you plan to tell about your plan to take PrEP:

Q14.

If you told these people, do you think their reaction will be supportive?	YES	NO	N/A
	▼	▼	▼
a. Your main male partner	1	2	99
b. Your other sex partners	1	2	99
c. Your mother	1	2	99
d. Your father	1	2	99
ex. Your sister or brother	1	2	99
i. Friends	1	2	99
jx. Other persons	1	2	99

Q15. Would your main male partner support your PrEP use?

Yes ❑1 **[GO TO Q17]**
No ❑2
N/A ❑99 **[GO TO Q17]**

Q16. Would your main male partner keep you from using PrEP?

Yes ❑1
No ❑2

Q17. Where do you think you will get the most support for using PrEP? Who offers the most support?

Clinicians	❑1
Friends	❑2
Family	❑3
Sexual partners (any)	❑4
Someone else	❑99

Please specify who else offers the most support for using PrEP:______________________

SECTION R: HOPE FOR THE FUTURE

Now we would like to ask you some questions about your future and your hope for success in your life. Please tell us how much you agree with the following statements about yourself:

R1.

	Strongly disagree	Disagree	Agree	Strongly agree
a) I know that my life will be better in the future.	❑1	❑2	❑3	❑4
b) The important people in my life tell me that I will have a successful life.	❑1	❑2	❑3	❑4
c) I trust that I will achieve the goals that I set for myself.	❑1	❑2	❑3	❑4
d) I believe that I will be successful even when there are difficulties in my life now.	❑1	❑2	❑3	❑4
e) I believe that the things I am doing now are preparing me for what I want in the future.	❑1	❑2	❑3	❑4
f) I can achieve my dreams if I focus on them.	❑1	❑2	❑3	❑4

This completes our interview. We know this has been very long and we really appreciate your time and patience. We hope you will return for the next interview. It will be much shorter. Thank you for participating in this important study.

END TIME:...|__|__| : |__|__| **(Use 24-hr clock)**
