# Supplementary material for: The PrEPARE Pretoria Project: protocol for a cluster-randomized factorial-design trial to prevent HIV with PrEP among adolescent girls and young women in Tshwane, South Africa
Source: BMC Public Health. 2020 Sep 15;20:1403. doi: 10.1186/s12889-020-09458-y (PMC7490774; doi:10.1186/s12889-020-09458-y)
Supplement: Supplementary file 5 — Additional file 5: Supplementary File 4. Estimated Detectable Differences in PrEP Uptake between AGYW in the PrEP/SRH + YWHC arm vs. PrEP/SRH only, controlling for intervention arm of clinic. [file 12889_2020_9458_MOESM5_ESM.pdf]

Supplementary Table. Estimated Detectable Differences in PrEP Uptake between AGYW in the PrEP/SRH+YWHC arm vs. PrEP/SRH only, controlling for intervention arm of clinic

Proportion of AGYW who initiate PrEP in PrEP/SRH only Arm	Proportion of AGYW who initiate PrEP in PrEP/SRH+YWHC Arm	Difference
20%	31%	11%
30%	42%	12%
40%	53%	13%
50%	63%	13%
10%	19%	9%
