# Supplementary material for: The PrEPARE Pretoria Project: protocol for a cluster-randomized factorial-design trial to prevent HIV with PrEP among adolescent girls and young women in Tshwane, South Africa
Source: BMC Public Health. 2020 Sep 15;20:1403. doi: 10.1186/s12889-020-09458-y (PMC7490774; doi:10.1186/s12889-020-09458-y)
Supplement: Supplementary file 7 — Additional file 7: Supplementary File 6. Ethical approval proofs. [file 12889_2020_9458_MOESM7_ESM.pdf]

THE SOUTH AFRICAN MEDICAL ASSOCIATION
Tel: +27 (0)12 481 2000 | Fax: +27 (0)12 481 2100 | www.samedical.org
Block F | Castle Walk Office Park | Nossob Street | Erasmuskloof | Ext 3 | Pretoria | 0181
PO Box 74789 | Lynnwood Ridge | 0040
Reg No 1927/000136/08 | NPC

11 January 2019

RTI International
3040 Cornwallis Road
PO Box 12194
Research Triangle Park
NC 27709
2194
USA

Attention: Jackie Ndirangu
jndirangu@rti.org

Dear Jackie

FINAL APPROVAL

**Protocol Number:**	**0216102**
**Protocol Title:**	The PrEPARE Pretoria Project: Prevention, Empowering, and Protecting Young Women in South Africa.

Your application dated 11 November 2018 for ethical evaluation of the abovementioned study was considered by the South African Medical Association Research Ethics Committee, at its meeting held on 28 November 2018.

The Ethics Committee is a registered IRB at the Office of Human Research Protection of the USA. Federal Wide Assurance was also received from the Office of Human Research Protection (Office of Human Research Protection –Group).

SAMAREC follows the standards adopted by the latest version of the American Food & Drug Administration (FDA) and ICH Harmonized Tripartite Guidelines for Good Clinical Practice; and conforms to the guidelines laid down by the World Medical Association, in particular, the Declaration of Helsinki (October 2013), the Belmont Report, the National Department of Health and the SA Medical Research Council.

The following members were present at the meeting:

Prof J R Snyman<br>MBChB, M Pharm Med, MD	Male, Pharmacologist, SAMA Member
Dr M Groenewald<br>MBChB, DCH (SA), PG Dip Int Res Ethic	Female, General Practitioner, SAMA Member
Prof M Kakaza<br>MBChB, Mmed	Female, Specialist Neurologist, SAMA Member
Mrs B Fineberg	Female, Educator

BSc (Hons); H.D.E	
Ms U Behrtel<br>BLC.LLB,Cert MedLaw	Female, Attorney
Dr S Franszen<br>MBCH.B, M.Med (O & G) , ACAM (Chel), BA (Theo)	Female, General Practitioner, SAMA Member
Ms W Massangaie<br>LLB	Female, Attorney
Mr M le Roux<br>B.Th, M.Div	Male, Legal Secretary
Dr T Lengana<br>MBBCh, FCNP (SA), MSc Med (Bioethics & Health Law)	Male, Specialist Nuclear Physician, SAMA Member

Resolution
After noting and considering the input of the evaluators on the above-mentioned study, the committee **RESOLVED** that the application be **approved.**

1. COVERING LETTER

The Covering Letter, dated 16 November 2018 was noted and accepted.

The request to include 16 year and older participants and the request to waiver Parentis Consent has been approved due to the following reasons:

i) This specific study design is not invasive and merely a collection of data
ii) The Benefit / Risk assessment clearly shows more benefit
ii) Based on the South African Department of Health Ethics in Health Research Principles, Process and Structures (2015):
"3.2.2.4 Minors' independent consent
In particular circumstances, e.g. for reasons of sensitivity, like discussion about sexual activities, substance abuse etc. it may be desirable and ethically justifiable for minors (especially older minors i.e. 16 years and older) to choose independently i.e. without parental assistance whether to participate in research. Generally, only minimal risk research is suitable for independent consent by minors. Reasons supporting the desirability of independent consent may include recruiting sufficient numbers of minors who otherwise would be unwilling to participate if they must tell their parents about the nature of the research in order to obtain parental permission."

2. ANNEXURE 1 - CLINIC AUDIT

Annexure 1 – The Clinic Audit was noted and approved.

3. ANNEXURE 2 - CLINICAL PROVIDER QUESTIONNAIRE

Annexure 2 – The Clinical Provider Questionnaire was noted and approved.

4. ANNEXURE 3 – SUPPORT STAFF QUESTIONNAIRE

Annexure 3 – The Support Staff Questionnaire was noted and approved.

5. ANNEXURE 4a – CLINICAL STAFF CONSENT_SD TRAINING

Annexure 4a – Clinical Staff Consent_SD Training Version 12/12/2018 was noted and approved.

6. **ANNEXURE 4b - CLINIC STAFF CONSENT_CONTROL**

Annexure 4b – Clinic Staff Consent_Control Version 12/12/2018 was noted and approved.

7. **ANNEXURE 5a PREPAREPRETORIAMARKETINGFLYER_REDUCED**

Annexure 5a – PrEParePretoriaMarketingFlyer_Reduced was noted and approved.

8. **ANNEXURE 5b PREPAREPRETORIA MARKETINGCARD_REDUCED**

Annexure 5b – PrEParePretoria-MarketingCard_Reduced was noted and approved.

9. **ANNEXURE 6 PREPAREPRETORIABROCHURE_REDUCED**

Annexure 6 – PrEParePretoriaBrochure_Reduced was noted and approved.

10. **ANNEXURE 7.SCREENER**

Annexure 7 – The Screener was noted and approved.

11. **ANNEXURE 8. RELEASE FORM FOR MEDICAL RECORDS**

Annexure 8 – The Release Form for Medical Records was noted and approved.

12. **ANNEXURE 9 - CONSENT FOR HIV AND PREGNANCY VERIFICATION TESTING**

Annexure 9 – Consent for HIV and Pregnancy Verification Testing Version 12/12/ 2018 was noted and approved.

13. **ANNEXURE 10 - BIOLOGICAL_VERIFICATION OF ELIGIBILITY**

Annexure 10 – The Biological_Verification of Eligibility was noted and approved.

14. **ANNEXURE 11 - APPOINTMENT CARD**

Annexure 11 – The Appointment Card was noted and approved.

15. **ANNEXURE 12 - AGYW CONSENT-ASSENT**

Annexure 12 – AGYW Consent Assent Version 17 December 2018 was noted and approved.

16. **ANNEXURE 13 - CONSENT CHECKLIST_CLINICIAN**

Annexure 13 – Consent Checklist_Clinician was noted and approved.

17. **ANNEXURE 14 - PARENTAL OR IN LOCO PARENTIS CONSENT**

The request for Parental or in Loco Parentis Consent is waived – kindly see rationale under 1. Covering Letter.

18. ANNEXURE 15 - PARENTAL OR IN LOCO PARENTIS CONFIDENTIALITY AGREEMENT FORM

The request for Parental or in Loco parentis Consent is waived – kindly see rationale under 1. Covering Letter.

19. ANNEXURE 16 - WORKSHEET D FOR RESEARCH WITH CHILDREN

Annexure 16 – The Worksheet D for Research with Children was noted and approved.

20. ANNEXURE 17 - RIGHTS OF RESEARCH PARTICIPANTS

Annexure 17 – The Rights of Research Participants was noted and approved.

21. ANNEXURE 18 – LOCATOR FORM

Annexure 18 – The Locator Form was noted and approved.

22. ANNEXURE 19 - PREPARE BASELINE QUESTIONNAIRE_NOV 15 2018

Annexure 19 – PrEPARE Baseline Questionnaire_Nov 15 2018 was noted and approved.

23. ANNEXURE 20 - BIOLOGICAL FORM

Annexure 20 – The Biological Form was noted and approved.

24. ANNEXURE 21 - PARTICIPANT SATISFACTION FORM

Annexure 21 – The Participant Satisfaction Form was noted and approved.

25. ANNEXURE 22 - REFERRAL FORM

Annexure 22 – The Referral Form was noted and approved.

26. ANNEXURE 23 - RESOURCE GUIDE

Annexure 23 – The Resource Guide was noted and approved.

27. ANNEXURE 24. STAFF CONFIDENTIALITY

Annexure 24 – The Staff Confidentiality was noted and approved.

28. ANNEXURE 25 - PROCEDURES FOR HANDLING DISTRESSED PARTICIPANTS

Annexure 25 – The Procedures for Handling Distressed Participants was noted and approved.

29. ANNEXURE 26. INCIDENT REPORT

Annexure 26 – The Incident Report was noted and approved.

30. CURRICULUM VITAE OF PRINCIPAL INVESTIGATORS AND OTHER STUDY STAFF

The Committee noted the Investigators CVs, declarations and other relevant documentation and RESOLVED as follows:

Site Address	Investigator	CV, Declaration and other supporting documents	Status
**Research Triangle Park 3040 East Cornwallis Road NC 27709-2194**	**Dr W Wechsberg Principal Investigator**	Noted and accepted.	Approved.
	**Dr A Minnis Sub Investigator**	Noted and accepted.	Approved.
	**Dr L Nyblade Sub-Investigator**	Noted and accepted.	Approved.
	**Dr F Browne Sub-Investigator**	Noted and accepted.	Approved.
	**Dr I Speizer Sub-Investigator**	Noted and accepted.	Approved.
	**Dr K Ahmed Sub-Investigator**	Noted and accepted.	Approved.
	**Dr B Myers Sub-Investigator**	Noted and accepted.	Approved.
	**Dr C Bonner Sub Investigator**	Noted and accepted.	Approved.
	**Ms J Ndirangu Project Director**	Noted and accepted.	Approved.

- *Kindly take note that all health professionals who dispense medicines are required to have a dispensing licence, unless a pharmacist is on site to dispense such medicines. In addition, medical practitioners with dispensing licenses are only permitted (legally) to dispense on their own prescriptions*
- *Kindly ensure that GCP Training is renewed in due course*
- *Take note that Pharmacists dispensing medicines require Malpractice Insurance*
- *Kindly ensure that expired registrations with Statutory Councils must be updated/renewed as appropriate*
- *Clinical trial information on CV's should have the names-of sponsors, abbreviated titles and protocol numbers of all studies listed.*
- *Kindly take note that all registered nurses who will be performing duties which fall within the scope of the nursing profession, are required to hold professional indemnity insurance in their personal capacities, unless their professional actions will be indemnified / covered by the insurance held by the sponsor. The onus is on the sponsor to ensure that the necessary and adequate insurance and professional indemnity coverage is in place, before commencement of the study.*

31. SAMAREC PROTOCOL_11.15

The SAMAREC Protocol_11.15 was noted and approved.

Yours Sincerely

Adri van der Walt
SAMAREC Officer
SA Medical Association
Tel: (012) 481 2046
samarec@samedical.org

IRB Authorization Agreement

Name of Research Project: The PREPaRE Project: PRevention, Empowering and PRotEcting Young Women in South Africa
Principal Investigator(s): Wendee Wechsberg
IRB Protocol Number or Project Number: SAMAREC: 0216102 RTI: 14142
Sponsor or Funding Agency: NICHD
Award Number, if any: 1R01HD094629.01

Name of Institution Providing IRB Review (Institution A): The South African Medical Association
OHRP Assurance Number (MPA or FWA): FWA00027240
IRB Registration Number: IRB00011624

Name of Institution Relying Upon IRB Review Above (Institution B): RTI International
OHRP Assurance Number (MPA or FWA): FWA00003331
IRB Registration Numbers: IRB00000653, IRB00000655

The Officials signing below agree that Institution B may rely on the above IRB review, approval, and continuing oversight provided under Institution A's Assurance for the above named research study.

The review, approval, and continuing oversight performed by the IRB satisfy the requirements of the HHS regulations for the protection of human subjects at 45 CFR 46, as well as the requirements of Institution A's OHRP-approved Assurance.

Institution A will supply relevant minutes of IRB, copies of IRB review approvals, including initial reviews, any amendments and continuing reviews to Institution B upon request. In addition, Institution A will notify Institution B of any complaints, adverse events, serious adverse events and/or unanticipated problems involving risks to subjects or others. Also, Institution A will notify Institution B about any serious or continuing noncompliance in regard to this study, any reports made to the DHHS Office for Human Research Protection (OHRP), any suspension or termination of IRB approval; or any other important findings that affect the IRB approval of this research study. Institution B remains responsible for ensuring compliance with the IRB's determinations and with the terms of its OHRP-approved Assurance. Either institution may terminate this agreement at any time by providing written notice.

This document should be kept on file at both institutions and must be provided to OHRP upon request.

Signatures:

Authorized Official of Institution "A" [signature] 8 March 2019.

Prof J Snyman — **Date**
Chairperson
SA Medical Association

Authorized Official of Institution "B" [signature] 03/06/2019

Juesta Caddell, Ph. D. — **Date**
IRB Director
Office of Research Protection
RTI International

THE SOUTH AFRICAN MEDICAL ASSOCIATION
Tel: +27 (0)12 481 2000 | Fax: +27 (0)12 481 2100 | www.samedical.org
Block F | Castle Walk Office Park | Nossob Street | Erasmuskloof | Ext 3 | Pretoria | 0181
PO Box 74789 | Lynnwood Ridge | 0040
Reg No 1927/000136/08 | NPC

14 March 2019

RTI International
3040 Cornwallis Road
PO Box 12194
Research Triangle Park
NC 27709
2194
USA

Attention: Jackie Ndirangu
jndirangu@rti.org

Dear Jackie

AMENDMENT 1

**Protocol Number:**	**0216102**
**Protocol Title:**	The PrEPARE Pretoria Project: Prevention, Empowering, and Protecting Young Women in South Africa.

Your letter dated 28 February 2019 refers.

The Committee Member noted and approved the following received documentation:

- Annexure 1_Clinical Audit Form Final
- Annexure 4a_Clinic Staff SD_Participant Information and informed Consent Document.2.27.bnh
- Annexure 4b_Clinic Staff Control_Participant information and Informed Consent Document Consent.2.27.bnh
- Annexure 7_ Quick Field Screener Final
- Annexure 9_Consent for HIV and Pregnancy Status Verification Testing 2.27.bnh
- Annexure 10_Biologiclal Verification of Eligibility 2.27.bnh
- Annexure 12_Participant Information Informed Consent Assent Document 2.27.bnh
- Annexure 14_Mother.In Loco Parentis Informed Consent document Final
- Annexure 15_Mother or In Loco Parentis Confidentiality Agreement Form
- Annexure 19_Baseline Questionnaire Final
- Annexure 20_Biological Form Final
- Annexure 22_Referral Form Final
- Annexure 25_Procedures for Making Referrals for Distressed Respondents2.27.bnh
- SAMAREC Revised Protcol_Deferment 2.28

Yours Sincerely

Dr A Coetzee (Chairperson), Dr M Mzukwa (Vice-Chairperson), Prof. HM Coovadia (President), Dr M Thandrayen (General Manager)
South African Medical Association NPC
Reg. No: 1927/000136/08

Adri van der Walt
SAMAREC Officer
SA Medical Association
Tel: (012) 481 2046
samarec@samedical.org

THE SOUTH AFRICAN MEDICAL ASSOCIATION
Tel: +27 (0)12 481 2000 | Fax: +27 (0)12 481 2100 | www.samedical.org
Block F | Castle Walk Office Park | Nossob Street | Erasmuskloof | Ext 3 | Pretoria | 0181
PO Box 74789 | Lynnwood Ridge | 0040
Reg No 1927/000136/08 | NPC

01 April 2019

RTI International
3040 Cornwallis Road
PO Box 12194
Research Triangle Park
NC 27709
2194
USA

Attention: Jackie Ndirangu
jndirangu@rti.org

Dear Jackie

AMENDMENT 2

**Protocol Number:**	**0216102**
**Protocol Title:**	The PrEPARE Pretoria Project: Prevention, Empowering, and Protecting Young Women in South Africa.

Your letter dated 18 March 2019 refers.

The Committee Member noted and approved the following received documentation:

- Annexure 8 – Release Form for Medical Records 3.18.19
- Annexure 12_Participant information Informed ConsentAssent Document 3.18.19
- Annexure 14_Mother.In Loco Parentis Informed Consent Document 3.18.19
- Modification for PrEP 3.18.19

Yours Sincerely

AdWalt

Adri van der Walt
SAMAREC Officer
SA Medical Association
Tel: (012) 481 2046
samarec@samedical.org

Dr A Coetzee (Chairperson), Dr M Mzukwa (Vice-Chairperson), Prof. HM Coovadia (President), Dr M Thandrayen (General Manager)
South African Medical Association NPC
Reg. No: 1927/000136/08

04 July 2019

RTI International
3040 Cornwallis Road
PO Box 12194
Research Triangle Park
NC 27709
2194
USA

Attention: Jackie Ndirangu
jndirangu@rti.org

Dear Jackie

FOLLOW UP APPOINTMENT DOCUMENTS

**Protocol Number:**	**0216102**
**Protocol Title:**	The PrEPARE Pretoria Project: Prevention, Empowering, and Protecting Young Women in South Africa.

Your letter dated 26 June 2019 refers.

The Committee Member noted and approved the following documents:

- Annexure 1 – Participant Information Informed Consent Assent Document – Follow-up
- Annexure 2 - Clinic Staff Participant Information And Informed Consent Document Follow-up
- Annexure 3 – Participant Follow-up Questionnaire

Yours Sincerely

AdWalt

Adri van der Walt
SAMAREC Officer
SA Medical Association
Tel: (012) 481 2046
samarec@samedical.org

Dr A Coetzee (Chairperson), Dr M Mzukwa (Vice-Chairperson), Prof. HM Coovadia (President), Dr M Thandrayen (General Manager)
South African Medical Association NPC
Reg. No: 1927/000136/08

THE SOUTH AFRICAN MEDICAL ASSOCIATION

Tel: +27 (0)12 481 2000 | Fax: +27 (0)12 481 2100 | www.samedical.org
Block F | Castle Walk Office Park | Nossob Street | Erasmuskloof | Ext 3 | Pretoria | 0181
PO Box 74789 | Lynnwood Ridge | 0040
Reg No 1927/000136/08 | NPC

28 October 2019

RTI International
3040 Cornwallis Road
PO Box 12194
Research Triangle Park
NC 27709-2194
USA

Attention: Jackie Ndirangu
jndirangu@rti.org

Dear Jackie

STUDY FORM MODIFICATION

**Protocol Number:**	**0216102**
**Protocol Title:**	The PREPaRE Project: Prevention, Empowering, and ProtEcting Young Women in South Africa

Your letter dated 08 October 2019 refers.

The Committee Member noted and approved the following received document:

- Annexure 7 _ Quick Field Screener_10.07.19

Yours Sincerely

Adri van der Walt
SAMAREC Officer
SA Medical Association
Tel: (012) 481 2046
samarec@samedical.org

Dr A Coetzee (Chairperson), Dr M Mzukwa (Vice-Chairperson), Prof. HM Coovadia (President), Dr V Nhlapho (Acting General Manager)
South African Medical Association NPC
Reg. No: 1927/000136/08

THE SOUTH AFRICAN MEDICAL ASSOCIATION
Tel: +27 (0)12 481 2000 | Fax: +27 (0)12 481 2100 | www.samedical.org
Block F | Castle Walk Office Park | Nossob Street | Erasmuskloof | Ext 3 | Pretoria | 0181
PO Box 74789 | Lynnwood Ridge | 0040
Reg No 1927/000136/08 | NPC

29 November 2019

RTI International
3040 Cornwallis Road
PO Box 12194
Research Triangle Park
NC 27709-2194
USA

Attention: Jackie Ndirangu
jndirangu@rti.org

Dear Jackie

MINOR AMENDMENT NOVEMBER 2019

**Protocol Number:**	**0216102**
**Protocol Title:**	The PREPaRE Project: Prevention, Empowering, and ProtEcting Young Women in South Africa

Resolution
After noting and considering the input of the evaluators on the above-mentioned study, the committee **RESOLVED** that the application be **approved.**

1. **COVERING LETTER, dated 21 November 2019**
The Covering Letter, dated 21 November 2019 was noted and accepted.

2. **CLINIC STAFF CONTROL_PARTICIPANT INFORMATION AND INFORMED CONSENT DOCUMENT CONSENT_REVISED 11/21/19**
The Clinic Staff Control_Participant Information and Informed Consent Document Consent_Revised 11.21.19 was noted and approved.

3. **CLINIC STAFF SD_PARTICIPANT INFORMATION AND INFORMED CONSENT DOCUMENT_REVISED 11.21.2019**
The Clinic Staff SD_Participant Information and Informed Consent Document_Revised 11/21/2019 was noted and approved.

4. **CLINIC STAFF _ PARTICIPANT INFORMATION AND INFORMED CONSENT DOCUMENT – FOLLOW UP _ REVISED _ 11/21/19**
The Clinic Staff _ Participant Information and Informed Consent Document- follow up _ Revised _ 11/21/19 was noted and approved.

5. **PROTOCOL MODIFICATION FOR SURVEY_FINAL 11.21.19**
The Protocol Modification for Survey_Final 11.21.19 was noted and approved.

Dr A Coetzee (Chairperson), Dr M Mzukwa (Vice-Chairperson), Dr S Maweya (President), Dr V Nhlapho (Acting General Manager)
South African Medical Association NPC
Reg. No: 1927/000136/08

Yours Sincerely

Adri van der Walt
SAMAREC Officer
SA Medical Association
Tel: (012) 481 2046
samarec@samedical.org

THE SOUTH AFRICAN MEDICAL ASSOCIATION
Tel: +27 (0)12 481 2000 | Fax: +27 (0)12 481 2100 | www.samedical.org
Block F | Castle Walk Office Park | Nossob Street | Erasmuskloof | Ext 3 | Pretoria | 0181
PO Box 74789 | Lynnwood Ridge | 0040
Reg No 1927/000136/08 | NPC

05 February 2020

RTI International
3040 Cornwallis Road
PO Box 12194
Research Triangle Park
NC 27709-2194
USA

Attention: Jackie Ndirangu
jndirangu@rti.org

Dear Jackie

MINOR AMENDMENT January 2020

**Protocol Number:**	**0216102**
**Protocol Title:**	PrEPARE Pretoria Project: Prevention, Empowering, and Protecting Young Women in South Africa.

Your letter dated 09 January 2020 refers.

The Committee Member noted and approved the following documents:

- Annexure 27 Consent for Co-enrolment Check_12.02.19
- Annexure 28 Co-enrolment Check List & Entry Form_01.08.20
- Revised Protocol_01.08.20
- Annexure 2c _ Participant Information Informed ConsentAssent Document – 9mfollow up _ 01.09.20

Yours Sincerely

Adri van der Walt
SAMAREC Officer
SA Medical Association
Tel: (012) 481 2046
samarec@samedical.org

Dr A Coetzee (Chairperson), Dr M Mzukwa (Vice-Chairperson), Dr S Maweya (President), Dr V Nhlapho (Acting General Manager)
South African Medical Association NPC
Reg. No: 1927/000136/08

THE SOUTH AFRICAN MEDICAL ASSOCIATION
Tel: +27 (0)12 481 2000 | Fax: +27 (0)12 481 2100 | www.samedical.org
Block F | Castle Walk Office Park | Nossob Street | Erasmuskloof | Ext 3 | Pretoria | 0181
PO Box 74789 | Lynnwood Ridge | 0040
Reg No 1927/000136/08 | NPC

30 March 2020

RTI International
3040 Cornwallis Road
PO Box 12194
Research Triangle Park
NC 27709-2194
USA

Attention: Jackie Ndirangu
jndirangu@rti.org

Dear Jackie

STUDY ACTIVITIES MODIFICATION

**Protocol Number:**	**0216102**
**Protocol Title:**	The PREPaRE Project: Prevention, Empowering, and ProtEcting Young Women in South Africa

Your letter dated 20 March 2020 refers.

The Committee Member noted the modification to study activities due to the unprecedented COVID-19 pandemic.

Yours Sincerely

AFdWalt

Adri van der Walt
SAMAREC Officer
SA Medical Association
Tel: (012) 481 2046
samarec@samedical.org

THE SOUTH AFRICAN MEDICAL ASSOCIATION
Tel: +27 (0)12 481 2000 | Fax: +27 (0)12 481 2100 | www.samedical.org
Block F | Castle Walk Office Park | Nossob Street | Erasmuskloof | Ext 3 | Pretoria | 0181
PO Box 74789 | Lynnwood Ridge | 0040
Reg No 1927/000136/08 | NPC

10 April 2020

RTI International
3040 Cornwallis Road
PO Box 12194
Research Triangle Park
NC 27709
2194
USA

Attention: Jackie Ndirangu
jndirangu@rti.org

Dear Jackie

FOLLOW UP APPOINTMENT DOCUMENTS

**Protocol Number:**	**0216102**
**Protocol Title:**	The PrEPARE Pretoria Project: Prevention, Empowering, and Protecting Young Women in South Africa.

Your letter dated 31 March 2020 refers.

The Committee Member noted and approved the following documents:

- Annexure 1 – Support Staff Questionnaire_FU_03.31.20_Final
- Annexure 2 - Support Staff Questionnaire_SD FU_03.31.20_Final
- Annexure 3 – Support Staff Questionnaire_SD FU_03.31.20
- Annexure 4 – Clinical Staff Questionnare_ FU 03.31.20_Final
- Annexure 5 – Clinical Staff Questionnare_SD FU 03.31.20_Final
- Revised Protocol _Final 02.25.20

Yours Sincerely

AFdWalt

Adri van der Walt
SAMAREC Officer
SA Medical Association
Tel: (012) 481 2046
samarec@samedical.org

Dr A Coetzee (Chairperson), Dr M Mzukwa (Vice-Chairperson), Prof. HM Coovadia (President), Dr M Thandrayen (General Manager)
South African Medical Association NPC
Reg. No: 1927/000136/08

THE SOUTH AFRICAN MEDICAL ASSOCIATION
Tel: +27 (0)12 481 2000 | Fax: +27 (0)12 481 2100 | www.samedical.org
Block F | Castle Walk Office Park | Nossob Street | Erasmuskloof | Ext 3 | Pretoria | 0181
PO Box 74789 | Lynnwood Ridge | 0040
Reg No 1927/000136/08 | NPC

28 April 2020

RTI International
3040 Cornwallis Road
PO Box 12194
Research Triangle Park
NC 27709-2194
USA

Attention: Jackie Ndirangu
jndirangu@rti.org

Dear Jackie

STUDY ACTIVITIES MODIFICATION

**Protocol Number:**	**0216102**
**Protocol Title:**	The PREPaRE Project: Prevention, Empowering, and ProtEcting Young Women in South Africa

Your letter dated 22 April 2020 refers.

The Committee Member noted the modification to study activities due to the unprecedented COVID-19 pandemic.

Yours Sincerely

Adri van der Walt
SAMAREC Officer
SA Medical Association
Tel: (012) 481 2046
samarec@samedical.org

Dr A Coetzee (Chairperson), Dr M Mzukwa (Vice-Chairperson), Prof. HM Coovadia (President), Dr V Nhlapho (Acting General Manager)
South African Medical Association NPC
Reg. No: 1927/000136/08

THE SOUTH AFRICAN MEDICAL ASSOCIATION
Tel: +27 (0)12 481 2000 | Fax: +27 (0)12 481 2100 | www.samedical.org
Block F | Castle Walk Office Park | Nossob Street | Erasmuskloof | Ext 3 | Pretoria | 0181
PO Box 74789 | Lynnwood Ridge | 0040
Reg No 1927/000136/08 | NPC

28 April 2020

RTI International
3040 Cornwallis Road
PO Box 12194
Research Triangle Park
NC 27709-2194
USA

Attention: Jackie Ndirangu
jndirangu@rti.org

Dear Jackie

MINOR AMENDMENT COVID-19

**Protocol Number:**	**0216102**
**Protocol Title:**	The PREPaRE Project: Prevention, Empowering, and ProtEcting Young Women in South Africa

Resolution
After noting and considering the input of the evaluators on the above-mentioned study, the committee **RESOLVED** that the application be **approved.**

1. **COVERING LETTER, dated 23 April 2020**
   The Covering Letter, dated 23 April 2020 was noted and accepted.

2. **PREPaRE COVID QUESTIONNAIRE**
   The PREPaRE COVID Questionnaire dated 23 April 2020 was noted and approved.

Yours Sincerely

AdWalt

Adri van der Walt
SAMAREC Officer
SA Medical Association
Tel: (012) 481 2046
samarec@samedical.org

Dr A Coetzee (Chairperson), Dr M Mzukwa (Vice-Chairperson), Dr S Maweya (President), Dr V Nhlapho (Acting General Manager)
South African Medical Association NPC
Reg. No: 1927/000136/08
