# Supplementary material for: The PrEPARE Pretoria Project: protocol for a cluster-randomized factorial-design trial to prevent HIV with PrEP among adolescent girls and young women in Tshwane, South Africa
Source: BMC Public Health. 2020 Sep 15;20:1403. doi: 10.1186/s12889-020-09458-y (PMC7490774; doi:10.1186/s12889-020-09458-y)
Supplement: Supplementary file 7 [file 12889_2020_9458_MOESM1_ESM.docx]

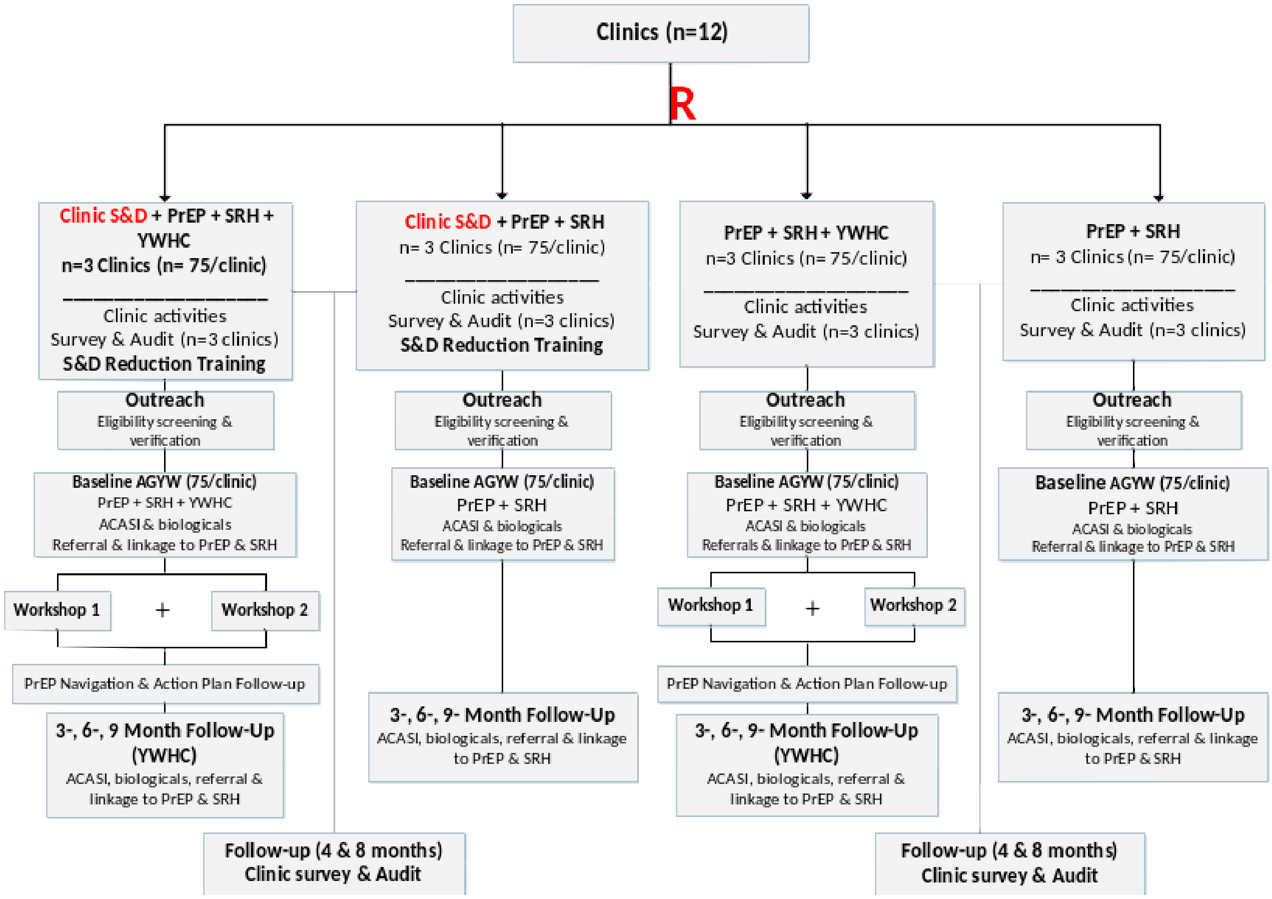

Clinics (n=12)
R
Clinic S&D + PrEP + SRH + YWHC
n=3 Clinics (n= 75/clinic)
Clinic activities
Survey & Audit (n=3 clinics)
S&D Reduction Training
Clinic S&D + PrEP + SRH
n= 3 Clinics (n= 75/clinic)
Clinic activities
Survey & Audit (n=3 clinics)
S&D Reduction Training
PrEP + SRH + YWHC
n=3 Clinics (n= 75/clinic)
Clinic activities
Survey & Audit (n=3 clinics)
PrEP + SRH
n= 3 Clinics (n= 75/clinic)
Clinic activities
Survey & Audit (n=3 clinics)
Outreach
Eligibility screening & verification
Baseline AGYW (75/clinic)
PrEP + SRH + YWHC
ACASI & biologicals
Referral & linkage to PrEP & SRH
Baseline AGYW (75/clinic)
PrEP + SRH
ACASI & biologicals
Referral & linkage to PrEP & SRH
Baseline AGYW (75/clinic)
PrEP + SRH + YWHC
ACASI & biologicals
Referrals & linkage to PrEP & SRH
Baseline AGYW (75/clinic)
PrEP + SRH
ACASI & biologicals
Referral & linkage to PrEP & SRH
Workshop 1
+
Workshop 2
PrEP Navigation & Action Plan Follow-up
3-, 6-, 9 Month Follow-Up (YWHC)
ACASI, biologicals, referral & linkage to PrEP & SRH
3-, 6-, 9- Month Follow-Up
ACASI, biologicals, referral & linkage to PrEP & SRH
3-, 6-, 9- Month Follow-Up (YWHC)
ACASI, biologicals, referral & linkage to PrEP & SRH
3-, 6-, 9- Month Follow-Up
ACASI, biologicals, referral & linkage to PrEP & SRH
Follow-up (4 & 8 months)
Clinic survey & Audit
Follow-up (4 & 8 months)
Clinic survey & Audit
